# Revision of the South American genus *Gaujonia* Dognin (Noctuidae, Pantheinae) with descriptions of five new genera and twenty-one new species

**DOI:** 10.3897/zookeys.985.51622

**Published:** 2020-11-05

**Authors:** Jose I. Martinez

**Affiliations:** 1 Florida Museum of Natural History, McGuire Center for Lepidoptera and Biodiversity, University of Florida, Gainesville, FL, 32611, USA, University of Florida Gainesville United States of America; 2 Entomology and Nematology Department, University of Florida, Gainesville, FL, 326011, USA University of Florida Gainesville United States of America

**Keywords:** Andean Mountains, DNA barcoding, host plants, jaguar moths, Neotropics, systematics, taxonomy

## Abstract

The endemic Neotropical genus *Gaujonia* Dognin is revised. Morphological characters and a phylogenetic analysis demonstrate paraphyletic relationships among the species. Four different groups are interpreted to represent four different genera. The *G.arbosi* group is the only remaining clade in the genus *Gaujonia*, and the other groups have been arranged into three new genera: *Millerana***gen. nov.**, *Oculicattus***gen. nov.**, and *Cicadoforma***gen. nov.** Additionally, two other genera *Cicadomorphus***gen. nov.**, and *Gaujoptera***gen. nov.** were found using morphological and molecular analyses based on some specimens that were misidentified as *Gaujonia* spp. A total of five new genera, three new combinations (*Cicadoformavau-nigrum* Hampson, **comb. nov.**, *Oculicattusrenifera* Hampson, **comb. nov.**, and *Milleranaarbosioides* Dognin, **comb. nov.**) and 21 new species (*Cicadoformaocelotus***sp. nov.**, *Cicadomorphuschicharra***sp. nov.**, *Cicadomorphuschuya***sp. nov.**, *Cicadomorphusfalkasiska***sp. nov.**, *Cicadomorphuslilianae***sp. nov.**, *Gaujoniabichu***sp. nov.**, *Gaujoniachiqyaq***sp. nov.**, *Gaujoniakanakusika***sp. nov.**, *Gaujoniasourakovi***sp. nov.**, *Gaujopteraamsa***sp. nov.**, *Milleranaaustini***sp. nov.**, *Milleranacajas***sp. nov.**, *Milleranacundinamarquensis***sp. nov.**, *Milleranamatthewsae***sp. nov.**, *Milleranatigrina***sp. nov.**, *Oculicattusboliviana***sp. nov.**, *Oculicattusbrehmi***sp. nov.**, *Oculicattusinca***sp. nov.**, *Oculicattusraizae***sp. nov.**, *Oculicattusschmidti***sp. nov.**, and *Oculicattusuturunku***sp. nov.**) are established.

## Introduction

*Gaujonia* Dognin (Lepidoptera: Noctuidae: Pantheinae) is a poorly studied Neotropical moth genus with a distribution restricted to the Andean Mountains, from Venezuela to Bolivia. Species of this genus are known as “jaguar moths” together with species from two other genera: *Bathyra* Walker and *Lichnoptera* Herrich-Schäffer. *Gaujonia* was based on the type species of *Gaujoniaarbosi* Dognin from Loja, Ecuador, which previously included three other species: *G.arbosioides* Dognin, *G.renifera* Hampson, and *G.vau-nigrum* Hampson. Seitz (1919–1944) illustrated the same specimens as Hampson (1913), with the only difference being the color rendition of *G.arbosi*. *Gaujonia* was not mentioned again in the literature until the check list of Poole (1989), and when Gara and Onore (1989) found that “*Gaujonia arbosi*” (actually, *Milleranatigrina* sp. nov. described herein) is a pest of pines in Ecuador.

After more than a century, and with the increasing use of molecular techniques in taxonomy, it is now possible to identify many new species worldwide, and *Gaujonia* species are no exception. This study is the first in a series of revisions of Neotropical Pantheinae following the works of G. G. Anweiler and B. C. Schmidt in North America (Anweiler 2009; Schmidt and Anweiler 2010, 2020) and G. Behounek, H. L. Han and V. S. Kononenko in Eurasia (Behounek and Kononenko 2011a, b; Behounek et al. 2011–2016). This study includes re-descriptions of the four known *Gaujonia* species and descriptions of five new genera and 21 new species. Additionally, three of the previously described *Gaujonia* species are transferred to new genera.

## Materials and methods

Genitalia preparation and terminology follow the protocols of Lafontaine (1987, 2004) and Schmidt and Anweiler (2020). Genitalia were stained with 10% eosin Y and examined in 30% ethanol. Only the genitalia from type specimens were mounted on slides using Euparal; the remainder were stored in vials of pure glycerin. Pinned adults were photographed prior to dissection using a camera Canon EOS Rebel T5i with a Canon EF 100 mm f/2.8 USM Macro lens. The genitalia were photographed following mounting employing a StackShot automated focus stacking macro rail with a camera Canon EOS 6D and an Infinity long-distance microscope Model K2 DistaMax.

Molecular diagnosis was performed by DNA barcoding, employing a segment from the mitochondrial cytochrome oxidase I (COI) gene for 29 species: one previously described species from *Gaujonia* sensu Hampson, 16 species newly described in this study, and the outgroup sequences of the rest of the jaguar moths along with a specimen from the type genus of *Gaujonia* (*Gaujoniaarbosi* Dognin) and two other unrelated genera of pantheines (*Meleneta* Smith and *Charadra* Walker) sequences were taken from the BARCODE OF LIFE DATA SYSTEM v4 (http://barcodinglife.com). DNA was extracted from leg tissue removed from pinned dry specimens, and Sanger sequencing was performed by the Canadian Centre for DNA Barcoding, Guelph, Ontario (http://ccdb.ca) following the protocols of Hebert et al. (2003). The sequences were concatenated and aligned using Geneious 9.1.3 (https://www.geneious.com). Phylogenetic trees were constructed using a maximum-likelihood (ML) analysis that was performed in IQ-TREE v. 2 to determine relationships among taxa following Nguyen et al. (2015), Hoang et al. (2018), and Minh et al. (2020). Branch support was estimated by performing 1000 replicates each for both ultrafast bootstraps (UFBoot2) (‘-bb’ command) and SH-aLRT test (SH-aLRT) (‘-alrt’ command). Phylogenetic relationships with UFBoot ≥ 95 and SH-aLRT ≥ 80 are considered to have strong support. Molecular data and photographs of the voucher specimens are available at BARCODE OF LIFE DATA SYSTEM v4 (project: Life History of Pantheinae). Sequences were submitted to GenBank (https://www.ncbi.nlm.nih.gov/genbank); accession numbers are listed in Suppl. material 1: Table S1.

Specimens were obtained from the following museums and collections:

## Systematics

An exhaustive revision of Neotropical pantheines employing morphological and molecular characters shows that *Gaujoniasensu* Dognin-Hampson is a multi-genus complex. The problem with *Gaujonia* (in the broad sense) is that the species have characters that subdivide them into four genera, as defined here:

**a) *arbosi* group (*Gaujonia*)**: represented by the type species of *Gaujonia*, *Gaujoniaarbosi*, and four new species *G.bichu* sp. nov., *G.chiqyaq* sp. nov., *G.kanakusika* sp. nov., and *G.sourakovi* sp. nov., in which male wings are entirely hyaline (transparent) with a minute line of scales on the veins and margins without orbicular or reniform spots; saccular and cucullar regions on the valva are separated; cucullar region squared and wide; aedeagus short; vesica long and wide, with two patches of thin setae; female wings hyaline with the line of scales wider than in male, with a small orbicular spot, but without a reniform spot; anal papilla wide; anterior apophysis small; appendix bursae and corpus bursae long and narrow, appendix bursae heavily sclerotized and smaller than corpus bursae.

**b) *arbosioides* group (*Millerana*)**: represented by the type *Milleranaarbosioides* misplaced originally in *Gaujonia*, and five new species: *M.austini* sp. nov., *M.cajas* sp. nov., *M.cundinamarquensis* sp. nov., *M.matthewsae* sp. nov., *M.tigrina* sp. nov., which differ from other groups in that the male antenna is serrate, whereas that of the female is filiform; wings are not hyaline; both orbicular and reniform spots are well developed; valva is simple with most species presenting an extension of the apex; aedeagus and vesica are narrow, with a band of spines surrounding the vesica near the base. Female genitalia are unknown.

**c) *renifera* group (*Oculicattus*)**: represented by the type species *Oculicattusrenifera* and seven new species: *O.boliviana* sp. nov., *O.brehmi* sp. nov., *O.inca* sp. nov., *O.raizae* sp. nov., *O.schmidti* sp. nov., and *O.uturunku* sp. nov; males have hyaline wings and veins covered by a narrow lines of scales; orbicular spot is barely visible; reniform spot is a long horizontal black line; valva with the saccular and cucullar regions separated; cucullar region long and narrow; aedeagus long; vesica long and narrow with one patch of thin setae near the base and two patches, one on each side of tip; female wings similar to those of male with orbicular spot large and line of scales wide; anal papillae narrow; anterior apophysis long; appendix bursae and corpus bursae long, similar in size.

**d) *vau-nigrum* group (*Cicadoforma*)**: includes the type species *Cicadoformavau-nigrum* and a new species *C.ocelotus* sp. nov. Male wings semi-hyaline (slightly translucent) with a large conspicuous orbicular spot and a small reniform spot; valva simple; aedeagus long; vesica with small patch of spines on upper side; female wings similar to those of male, but covered with scales; anal papilla wide; anterior apophysis long; appendix bursae small; corpus bursae broad.

**Figure 1.**
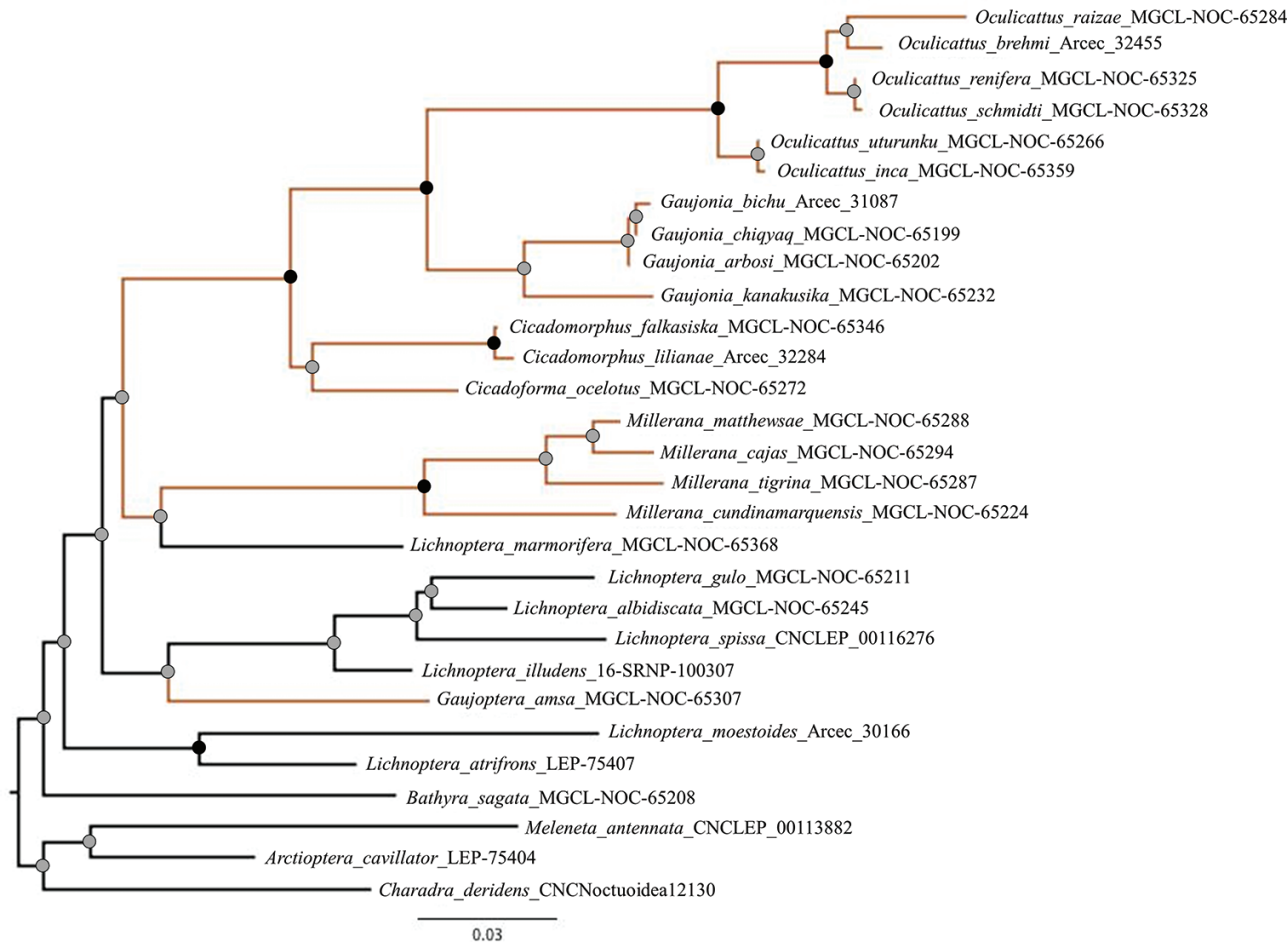
Maximum likelihood tree showing relationships between the *Gaujonia* genus group and the rest of the other jaguar moth genera based on the cytochrome c oxidase I gene (COI) marker. Nodes with black circles represent high support (UFBoot ≥ 95 and SH-aLRT ≥ 80). Nodes with gray circles represent low support (UFBoot < 95 and SH-aLRT < 80). Black branches constitute the outgroups.

Unfortunately, molecular data could not be obtained for seven of the species in the *Gaujonia* complex. However, the significant amount of morphological evidence presented in this study is sufficient to justify splitting *Gaujoniasensu* Dognin-Hampson into four genera, which is corroborated by the COI gene tree (Fig. 1).

### Key to the genera of the *Gaujonia* genus group based on adult male morphology

**Table:** 

1	Valva of male genitalia with clasper (Fig. 68)	***Gaujoptera***
–	Clasper absent in male genitalia (Figs 8–11, 59–67, 69–79)	**2**
2	Male genitalia with cucullar and saccular regions separated (Figs 8, 10, 64–67, 74–79)	**3**
–	Male genitalia with simple valva (Figs 9, 11, 59–63, 68–73)	**4**
3	Antenna brownish orange; eye with copper interfacetal setae; orbicular spot small; reniform spot with large lunate marking; genitalia with cucullar region narrow; apex rounded; aedeagus long and narrow; basal area of vesica with linear patch of spines, and two other polygonal patches mesally (Figs 7, 10, 43–53, 72–79)	***Oculicattus***
–	Antenna dark brown; interfacetal setae black; orbicular spot small, sometimes barely evident; a small dot in base of cell M1 instead of reniform spot; cucullus wide with apex flattened; aedeagus wide and short; vesica with oval patches of spines only in mesal area (Figs 4, 8, 26–31, 64–67)	***Gaujonia***
4	Antenna filiform, dark brown; forewing hyaline or semi-hyaline; cucullar area with a lower lobe separate from sacculus; vesica wider than aedeagus, with a wide band of small spines on its upper side (Figs 11, 12–19, 59–63)	**5**
–	Antenna serrate, dark brown or brownish orange; forewing covered by scales; lobe not separated from saccular area; vesica elongate with a narrow band of scattered spines surrounding vesica near base; sometimes a small patch of spines at terminal end of vesica (Figs 9, 35–42, 69–73)	***Millerana***
5	Forewing hyaline with scales only on margins and veins; line pattern only visible from posterior margin; genitalia with remarkably wide apex and lobe; valva with outer margin presenting a series of small indentations; vesica long with patch of spines on upper side and a band of spines near base (Figs 11, 12–15, 59)	***Cicadoforma***
–	Forewing semi-hyaline with scattered scales allover; line pattern visible, sometimes blurry; genitalia with apex and lobe distinctively narrow; valva without indentations; vesica rounded with only a patch of spines on upper side (Figs 16–19, 60–63)	***Cicadomorphus***

#### Cicadoforma gen. nov.

Taxon classificationAnimaliaLepidopteraNoctuidae

FC9BE9CA-6F49-51E2-ABAF-35E5710C1519

http://zoobank.org/725B4F17-CE86-4BE2-B01B-60C8EF82BED8

##### Gender.

Feminine.

##### Type species.

*Gaujoniavau-nigrum* Hampson, 1913. Catalogue of Lepidoptera Phalaenae in the British Museum 13: 385, 387, pl. 235, fig. 3.

##### Etymology.

*Cicadoforma* refers to how people in South America confuse this group with cicadas.

##### Included species.

The genus *Cicadoforma* is established to accommodate *C.vau-nigrum*, which was previously included in *Gaujonia* because of wing pattern similarities; a new species is described in *Cicadoforma*: *C.ocelotus* sp. nov. However, there are remarkable differences in morphology and molecular characters, as shown here.

##### Diagnosis.

*Cicadoforma* is similar to *Cicadomorphus*, not only externally, but internally as well; however, the phylogenetic analysis results showed enough evidence to separate *Cicadoforma* in a different genus from *Cicadomorphus* (Fig. 1). Nevertheless, both genera can be distinguished morphologically by the forewing, which is hyaline with scales only on margins and veins in *Cicadoforma*, whereas in *Cicadomorphus* has scales are more widely distributed on the forewing. Genitalia have some small indentations on outer margin of valva that are not present in *Cicadomorphus*; apex and lobe on valva is much wider in *Cicadoforma*; upper side of vesica with one patch of spines, and a narrow band of spines near the base are present in *Cicadoforma*. Female genitalia with square-shaped anal papillae in *Cicadoforma*; more rounded in *Cicadomorphus*. DNA barcodes show a closer relationship with *Cicadomorphus* (~ 5% divergent than with *Gaujonia* (~ 6%).

##### Description.

Sexually dimorphic mainly in size, female slightly larger than male; forewing in male with some hyaline areas and with poorly developed pattern, whereas female forewing semi-hyaline with pattern better defined. Antenna filiform, black, or dark brown in both sexes; antenna with yellow basal line of scales; eye large, covered by long interfacetal setae; palp with black upper side and yellow underside; haustellum dark brown and reduced, but functional. Forewing with orbicular reniform spots small. Hindwing semi-hyaline with scales only on margin and veins. Male genitalia with simple, lightly-sclerotized valva; valva wide apically with some small indentations on outer margin; clasper absent, cucullus wide, with a broad lobe extended in front of sacculus, ear-like in shape; sacculus with a small foot-like process; uncus crooked, long and wide; aedeagus short, with a simple long vesica with a broad patch of spines on upper side and a narrow band of spines at base. Female genitalia with a large square-shaped and lightly sclerotized sterigma, and a rugose, sclerotized appendix bursae; corpus bursae unsclerotized.

##### Immature stages.

Unknown.

##### Biology.

Unknown.

### Key to species of the genus *Cicadoforma* based on adult male morphology

**Table:** 

1	Thorax with small black dots; forewing with pattern well developed; orbicular spot large; vesica with spines close to base (Figs 11, 12)	***C.vau-nigrum***
–	Thorax with large black dots; pattern on forewing barely visible; orbicular spot small; basal area of vesica without spines (Figs 13–15, 59)	***C.ocelotus***

#### Cicadoforma ocelotus sp. nov.

Taxon classificationAnimaliaLepidopteraNoctuidae

A1529BC6-38E6-586E-AC5C-F4FBD6407849

http://zoobank.org/B8926D26-10E1-434D-B5B5-0CBED8A385CE

Figs 2 , 13–15 , 21 , 59 , 80 , 92

##### Type material.

***Holotype*** ♂, **Colombia**: Colombia, Santander road Duitama-Charala, 5°58'13’’N, 73°10'07’’W, 15–17.III.2016, 2900 m, leg Sinyaev & Machado, coll. Dr. Ron Brechlin / UF, FLMNH, MGCL 1049088. [DNA voucher MGCL-NOC-65272] deposited in MGCL. ***Paratypes*** (6 ♂, 2 ♀, MGCL): **Colombia**: same collecting data as holotype (1 ♂, 1 ♀); Colombia, Boyacá Arcabuco, Vereda Peñas Blancas, 2670 m, 5°47'05’’N, 73°26'17’’W, 18–22.II.2015; Sinyaev, M. Márquez & J. Machado, coll. Dr. Ron Brechlin (1 ♂); Colombia, Boyacá Arcabuco, Vereda Peñas Blancas, 2670 m, 5°47'05’’N, 73°26'17’’W, 20–22.IV.2015; Sinyaev, M. Márquez & J. Machado, coll. Dr. Ron Brechlin (1 ♂); Colombia, Boyacá Provincia del Norte road 55 Susacon – Santa Rosita, 3050 m, 6°10'40"N, 72°43'37"W, 27–28. IV.2017, V. Sinyaev (2 ♂); Colombia, Quindío W of Salento, 1950 m, 4°38'25"N, 75°34'46"W, 9–10.III.2017, V. Sinyaev (1 ♂, 1 ♀).

##### Etymology.

The species name *ocelotus* is derived from the characteristic yellowish orange coloration on its body, reminiscent of the color of an ocelot.

##### Diagnosis.

*Cicadoformaocelotus* can be distinguished from its only congener, *C.vau-nigrum*, by its remarkably brighter ground color, its larger orbicular spot, and shorter space between forewing veins R3 and R4. The valva of the male genitalia of *C.ocelotus* have concave outer margins, in contrast with the straight margin of *C.vau-nigrum*.

##### Description.

***Head*.** Palp short with a combination of black, yellow, and orange scales; ground color of frons orange or yellow with scattered black hair-like scales; antenna dark brown. ***Thorax*.** Yellow or orange with small spots scattered throughout dorsum. ***Wings*.** Forewing length: male 19–21 mm; female 25–27 mm; forewing yellow or orange scales covering veins and margins with semi-hyaline areas between them; subterminal line slightly visible; basal, antemedial, medial, and postmedial lines poorly developed, present only as small black dots on veins; reniform spot narrow with top and bottom surrounded by black scales and a small black dot in middle; orbicular spot small, elongated; female with antemedial, medial, postmedial, and subterminal lines slightly defined; reniform and orbicular spots similar to those of male; R4 almost entirely black; black V-shaped marking at base of CuA2; hindwing semi-hyaline with yellow or orange veins paler than forewing; fringe composed of short yellow or orange hair-like scales except on posterior margin, which has long, pale-yellow scales. ***Leg.*** Yellow or orange with some irregular brown spots that decrease in number from prothoracic legs to metathoracic legs. ***Abdomen*.** Covered by brownish orange scales that are paler than those of thorax; brown tufts in A2–A7 with tips with same color as remainder of abdomen. ***Male genitalia*.** Cucullus wide, lobe narrow; lobe apex rounded; apex clothed with short setae that expand over entire costal margin; sacculus base narrow; saccus relatively long, V-shaped; tegumen fairly flat; juxta U-shaped on upper side; aedeagus 3 ¾ × longer than wide with opening to vesica same width as aedeagus; vesica 1 ½ × longer than aedeagus with a patch of spines on upper side with some conspicuous basal spines. ***Female genitalia*.** Anal papilla wide, petal shaped, clothed with relatively long setae; A8 membranous, relatively short; posterior apophysis almost same length as anal papilla; sterigma enlarged, lightly sclerotized, fused above ostium; anterior apophysis ⅞ × shorter than posterior apophyses; ductus bursae short, strongly sclerotized and wide; posterior ¾ of appendix bursae strongly sclerotized, remainder including corpus bursae unsclerotized; corpus bursae 1 ¼ × longer than the appendix bursae.

##### Immature stages.

Unknown.

##### Distribution.

This species is only known from northern Colombia from moderate elevations from 2000–3000 m, especially in cloud forests (Fig. 92).

##### Biology.

Unknown.

##### Remarks.

This species is the only one in the genus group that has two different phenotypic variations, a yellow form, and an orange form, possibly due to elevation or seasonality (Figs 13–15, 21).

**Figures 2–7.**
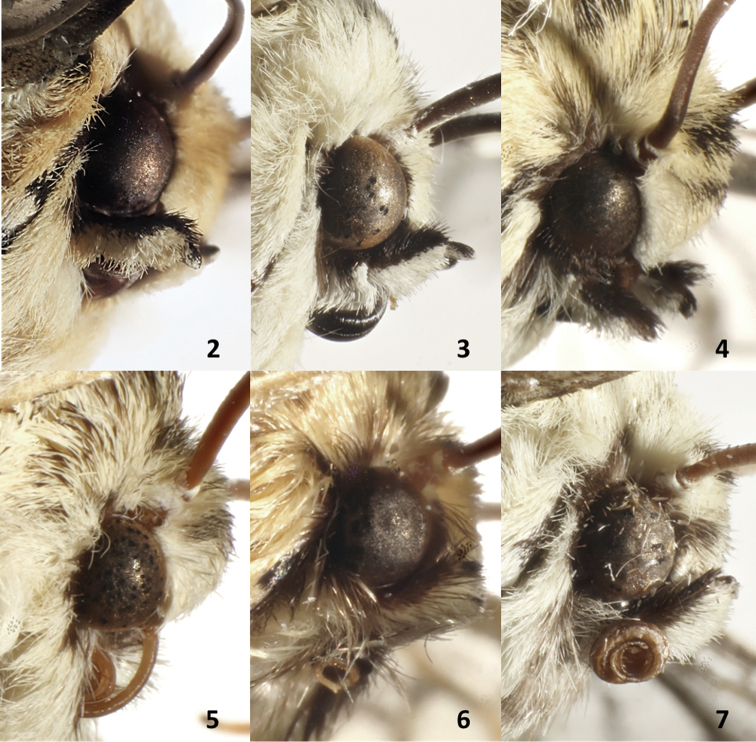
Adult head structure **2***Cicadoformaocelotus*, MGCL, Santander, Colombia **3***Cicadomorphusfalkasiska*, MGCL, Pasco, Peru **4***Gaujoniakanakusika*, MGCL, Cundinamarca, Colombia **5***Gaujopteraamsa*, MGCL, Cotapata, Bolivia **6***Milleranacajas*, MGCL, Azuay, Ecuador **7***Oculicattusrenifera*, MGCL, Cusco, Peru.

#### Cicadoforma vau-nigrum

Taxon classificationAnimaliaLepidopteraNoctuidae

(Hampson) comb. nov.

30BBF736-A092-5DA4-A95B-EF2BFB8BBFA3

Figs 11 , 12 , 20 , 81 , 92

Gaujonia vau-nigrum Hampson, 1913: 387 pl. 235, fig. 3.

##### Type material.

***Holotype***, ♂, **Venezuela**: “*Gaujonia vau.nigrum* type ♂ Hmpsn / Tovar, Venezuela Moritz / 1901-57 / Tovar Venezuela / Noctuidae ♂ Slide genitalia No. 5207 / NHMUK 010917654”, coll. G.Hampson, deposited in NHMUK. **Additional examined specimens** (8 ♂, 4 ♀, CUIC): **Venezuela**: Venezuela, Route Caracas-Colonia, Tovar, D. F.,IX.1939, Rene Lichy (1 ♂); Venezuela, El Junquito, D. F., 3 Apr. 1943, coll. Rene Lichy (1 ♂); no locality given, “j.15.X.44” / Franclemont diss. #5263 (1 ♂); no locality given, “j.22.VI.47” (1 ♂); no label data (2 ♀). (2 ♂, 1 ♀, CNC): **Venezuela**: same collecting data as the first CUIC male specimens (2 ♂); Same collecting data as the second CUIC male specimens (1 ♀).

##### Etymology.

George F. Hampson likely named this species “*vau-nigrum*” because of the black V-shaped mark at the base of CuA2.

##### Diagnosis.

*Cicadoformavau-nigrum* is the largest species in this genus; males have a forewing length of ca. 24–26 mm, whereas that of females is ca. 30–32 mm. This species is similar to *C.ocelotus*, but easy to distinguish by the size, and also by the orbicular spot, which is larger in *C.vau-nigrum*. Another important difference is that *C.vau-nigrum* males have hyaline wings where only the veins and margins are covered with yellow and black scales; the abdomen has dark brown tufts in the middle of A4–A7. *Cicadoformavau-nigrum* can be differentiated from other species by the male genitalia; the vesica has two patches of spines, one near to the aedeagus and the other on the upper side of the vesica. Female genitalia have heavily sclerotized ductus bursae and appendix bursae, with the corpus bursae ca. 2 × longer than the appendix bursae and 1½ × wider; the anterior apophyses are remarkably short, whereas the posterior apophyses are almost as long as the valva.

##### Immature stages.

Unknown.

##### Distribution.

*Cicadoformavau-nigrum* is endemic to Venezuela, known only from the states of Vargas and Aragua at moderate elevation in the northern part of the northwestern Andes (Fig. 92).

##### Biology.

Unknown.

##### Remarks.

The specimens that were examined were over 60 years old and DNA could not be obtained from them. The specimens from the CNC have rotting stains on the base of the wings, and one of the males is missing half of the abdomen. The only female has incomplete antennae.

**Figures 8–11.**
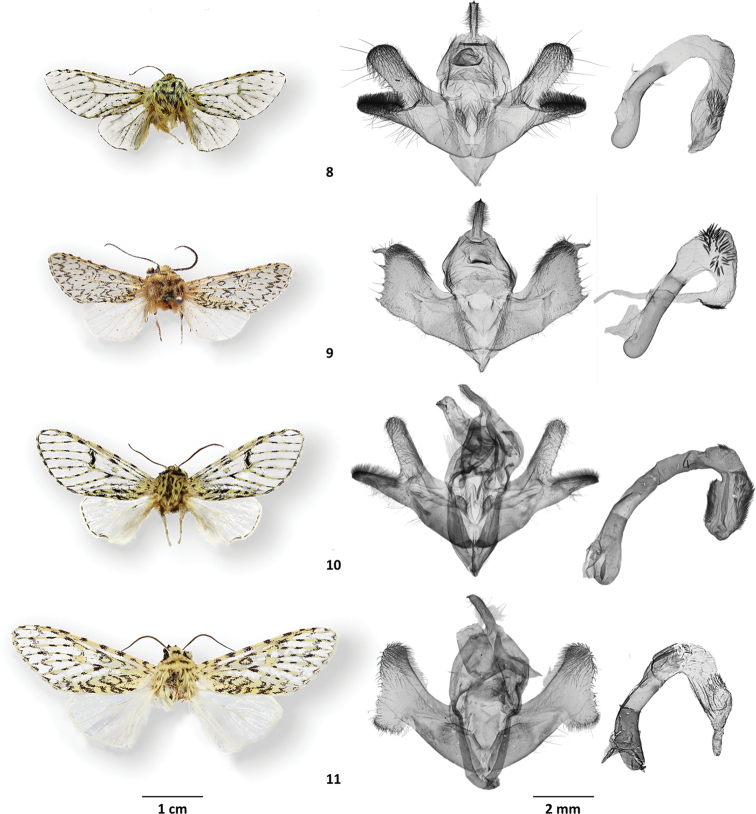
Type specimens **8***Gaujoniaarbosi*, lectotype, USNM, Loja, Ecuador **9***Milleranaarbosioides*, holotype, USNM, Loja, Ecuador **10***Oculicattusrenifera*, holotype, NHMUK, Puno, Peru **11***Cicadoformavau-nigrum*, holotype, NHMUK, Tovar, Venezuela.

#### Cicadomorphus gen. nov.

Taxon classificationAnimaliaLepidopteraNoctuidae

BAD2CC02-668F-547B-9957-85234CB2CFFA

http://zoobank.org/24926D2B-FFDA-41EC-8C37-1BAE0C3E0C84

##### Gender.

Masculine.

##### Type species.

*Cicadomorphuslilianae* sp. nov.

##### Etymology.

*Cicadomorphus* refers to how similar is this genus to *Cicadoforma*.

##### Included species.

The genus contains four new species, *Cicadomorphuschicharra* sp. nov., *Cicadomorphuschuya* sp. nov., *Cicadomorphusfalkasiska* sp. nov., and *Cicadomorphuslilianae* sp. nov., which were placed at first into *Cicadoforma*, but the molecular analyses place them as a sister group.

##### Diagnosis.

*Cicadomorphus* is closed related to *Cicadoforma* genetically and morphologically (see *Cicadoforma* diagnosis).

##### Description.

Sexually dimorphic in size, female slightly larger than male; cells of forewing with some semi-hyaline areas in male, whereas in female forewing covered by scales; line pattern conspicuous in both sexes. Antenna in both sexes dark brown, filiform with a stripe of sulfur-yellow scales basally; haustellum functional but reduced. Forewing with orbicular spot varying in size, reniform spot narrow. Hindwing semi-hyaline presenting scales only on margins and veins. Male genitalia with simple valva, lightly sclerotized lacking clasper; cucullar region with apex and ear-shaped lobe extremely narrow; saccular region with a long and narrow harpoon-shaped process; uncus hooked, broad and long; aedeagus short with simple vesica with a narrow patch of spines on upper side. Female genitalia with sterigma large, rounded, lightly sclerotized; appendix bursae rugose and sclerotized; corpus bursae transparent, not sclerotized.

##### Immature stages.

***Egg.*** Circular with soft surface with the chorion forming small square cells, which is consistent across the entire *Gaujonia* genus group. ***Larva*.** Passes through five to seven instars. Late instars with remarkably short secondary setae, which leave some spaces completely naked; they also present some scattered long setae especially on the prothorax, which cover the head capsule. ***Pupa*.** Covered by a dense cocoon. The three pair of legs are visible along with the antenna, but prothoracic legs are slightly concealed and micro-setae on the abdomen as in other pantheines, including in the other genera of the *Gaujonia* genus group.

##### Biology.

Currently known only for *Cicadomorphusfalkasiska* (see *C.falkasiska* diagnosis).

### Key to species of the genus *Cicadomorphus* based on adult male morphology

**Table:** 

1	Vesica gradually tapered toward apex (Figs 60, 62)	**2**
–	Vesica wide and rounded (Figs 61, 63)	**3**
2	Thorax and forewing whitish yellow; thorax with small dots; orbicular spot small; black tufts in middle of each segment on dorsal area of abdomen; valva with ventral lobe short (Figs 17, 60)	***C.chuya***
–	Thorax and forewing yellow; thorax with large black spots; orbicular spot large; A1 and A3 with yellow tuft; valva with ventral large and truncated (Figs 16, 62)	***C.lilianae***
3	Thorax and forewing greenish yellow; thorax densely covered with black dots dorsally; forewing with some hyaline areas with scales only on veins; valva with square-shaped apex; vesica with long narrow transverse band of spines (Figs 19, 63)	***C.chicharra***
–	Thorax and forewing pale yellow; thorax with few scattered black dots dorsally; forewing covered by scales; valva with apex rounded; vesica with short wide transverse band of spines (Figs 18, 61)	***C.falkasiska***

#### Cicadomorphus chicharra sp. nov.

Taxon classificationAnimaliaLepidopteraNoctuidae

79E999A1-528A-50C2-93CA-F1F65B70ECF1

http://zoobank.org/65303202-29DE-4933-A69C-D4943042E39A

Figs 19 , 63 , 92

##### Type material.

***Holotype*** ♂, **Bolivia**: Bolivia, La Paz, Santa Rosa de Lima, 16°23.6'S, 67°41.8'W, 20–22.10.2010, H = 1550 m, leg. Viktor Sinyaev & Oleg Romanov. Deposited in MGCL. ***Paratypes*** (2 ♂, MGCL): **Bolivia**: same collecting data as holotype.

##### Etymology.

The word *chicharra* means cicada in the Quechua language.

##### Diagnosis.

*Cicadomorphuschicharra* is one of the most easily identified species in the group due to its whitish yellow coloration, but also the wing pattern is thinner and paler than in related species, such as *C.lilianae*. The male genitalia have a wider vesica and narrower spine band on the upper side. Additionally, barcoding showed 2% divergence from *C.lilianae*.

##### Description.

***Head*.** Palp with last segment black with a small white dot; frons with greenish yellow scales; antenna brownish orange. ***Thorax*.** Greenish yellow, with small black dots dorsally; collar with margins black. ***Wings*.** Forewing length: male 23–25 mm; forewing greenish yellow, semi-hyaline areas clothed by fewer greenish yellow scales; subterminal, medial, and antemedial lines slightly visible; reniform spot poorly defined; orbicular spot long; hindwing: semi-hyaline with greenish yellow veins and fringe. ***Leg.*** Prothoracic legs black with some patches of same color as body; mesothoracic legs and metathoracic legs greenish yellow. ***Abdomen*.** Greenish yellow, paler than thorax; tufts in male on A1–A6 with characteristic small black spots on A1, A5 and A6. ***Male genitalia*.** Cucullus wide with a wide lobe; apex, outer margin, and lobe covered by long setae; apex slightly squared; sacculus wide with needle-shaped process; tegumen narrow, unsclerotized around uncus; saccus relatively wide; juxta with U-shaped concave depression on upper side; aedeagus ⅔ × as long as vesica; opening to vesica as wide as aedeagus; vesica wide with narrow transverse band of spines.

##### Immature stages.

Unknown.

##### Distribution.

The three specimens were found in the western zone of Bolivia at moderate elevations ca. 1500 m (Fig. 92).

##### Biology.

Unknown.

##### Remarks.

The holotype is in perfect condition (Fig. 19). Initially this species was confused with *Cicadomorphuslilianae*, but a rigorous morphological examination and DNA barcoding showed they were separate species.

**Figures 12–25.**
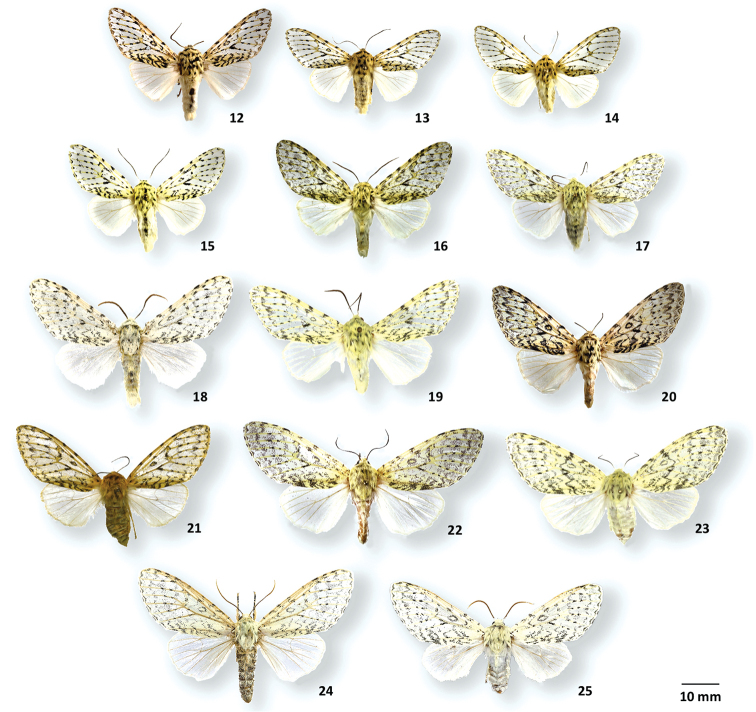
Adult habitus of *Cicadoforma* and *Cicadomorphus* species **12***Cicadoformavau-nigrum*, ♂, CNC, Tovar, Venezuela **13***C.ocelotus*, ♂, holotype, MGCL, Santander, Colombia **14***C.ocelotus*, ♂, paratype, MGCL, Boyacá, Colombia **15***C.ocelotus*, ♂, paratype, MGCL, Quindío, Colombia **16***Cicadomorphuslilianae*, ♂, holotype, MGCL, Napo, Ecuador **17***C.chuya*, ♂, holotype, MGCL, Tolima, Colombia **18***C.falkasiska*, ♂, holotype, MGCL, Oxapampa, Peru **19***C.chicharra*, ♂, holotype, MGCL, San Rosa, Bolivia **20***C.vau-nigrum*, ♀, CNC, El Junquito, Venezuela **21***C.ocelotus*, ♀, paratype, MGCL, Santander, Colombia **22***C.lilianae*, ♀, paratype, **FSU**, Zamora-Chinchipe, Ecuador **23***C.chuya*, ♀, paratype, MGCL, Cochabamba, Bolivia **24***C.falkasiska*, ♀, paratype, MGCL, Oxapampa, Peru **25***C.falkasiska*, ♀, paratype, MGCL, Junin, Peru.

#### Cicadomorphus chuya sp. nov.

Taxon classificationAnimaliaLepidopteraNoctuidae

B32B3358-D4A9-58C8-B875-2757B7002B27

http://zoobank.org/8A73E98E-2A87-4369-856C-5301675C5566

Figs 17 , 23 , 60 , 84 , 92

##### Type material.

***Holotype*** ♂, **Colombia**: Colombia, Tolima, Nevado del Tolima, 4°36'20’’N, 75°19'36’’W, 2850 m, 08–11.XII.2013, leg. Victor Sinyaev & Mildred Márquez / UF, FLMNH, MGCL 1049072, deposited in MGCL. ***Paratypes*** (5 ♂, 1 ♀, MGCL): **Colombia**: Colombia, Santander, km 23 rd. Barbosa – Arcabuco, 5°49'14’’N, 73°30'14’’W, 18–22.IX.2014, 2360 m, leg. V. Sinyaev & M. Márquez (1 ♂); **Peru**: Peru, Department Cuzco, Manu Park, San Pedro, 1800 m, III.1997, local people leg. (4 ♂); **Bolivia**: Bolivia, Cochabamba, Sierra Siberia, 16°48,3'S, 64°40,8'W, 01–02.XI.2010, H = 3060 m, leg. Viktor Sinyaev & Oleg Romanov (1 ♀).

##### Etymology.

*Ch’uya* means pure, transparent, or glassy in the Quechua language.

##### Diagnosis.

Although *Cicadomorphuschuya* shares some characters with *C.chicharra*, it is easy to distinguish by wing pattern; *C.chuya* is the only species that has both the terminal and subterminal lines serrated. In addition, the genitalia have the lobe reduced, and the juxta is pentagonal-shaped. The base of the lobe opens externally diagonally from the tornus to the cucullus.

##### Description.

***Head*.** Third segment of palp divided with upper side black, underside white, but white areas with a few brown spots; antenna dark brown basally, distal segments paler. ***Thorax*.** Whitish yellow coated with small black dots dorsally. ***Wings*.** Pattern in both sexes well defined and visible; forewing length: male 22–24 mm; female 29–31 mm; forewing whitish yellow with pattern somewhat blurred; nearly all lines visible and slightly blurred with exception of postmedial line, which is formed by black dots on wing veins plus some dispersed scales; subterminal and terminal lines zigzag; reniform spot relatively wide, outline poorly defined; orbicular spot medium sized and elongate; hindwing hyaline with yellow scales on fringe paler than thorax; wing veins darkened. ***Leg.*** Prothoracic and mesothoracic legs whitish yellow with two patches of brown scales on femur. ***Abdomen*.** Dorsally gray, black, and whitish yellow tufts in middle segment with A1 and A3 whitish yellow; whitish yellow ventrally. ***Male genitalia*.** Cucullar region wider close to apex; lobe small, almost completely covered by long setae from middle of costal margin to lobe; tooth-like protuberance barely visible; lobe diagonal to tornus of valva; saccular region relatively short with tip of process just touching tooth-like protuberance externally; saccus thin, rhomboid shaped; juxta pentagonal shaped; tegumen narrow with a small hood on base of uncus; aedeagus short and wide; opening to vesica square shaped ventrally; vesica tapered, with a large band of spines. ***Female genitalia***. Anal papilla square shaped with short setae; posterior apophysis almost same size as anal papilla; anterior apophysis short; sterigma V-shaped, lightly sclerotized, above ostium; ductus bursae wide posteriorly, narrow toward appendix bursae; appendix bursae ¼ × shorter than corpus bursae; corpus bursae semi-transparent.

##### Immature stages.

Unknown.

##### Distribution.

Males were found in Colombia and Peru, whereas the female was found in Bolivia, all in deciduous forest habitats at moderate to high elevations from 1800–3000 m (Fig. 92).

##### Biology.

Unknown.

##### Remarks.

The holotype is in near perfect condition (Fig. 17) with only the right hindwing slightly bent inwards at the posterior margin. The female is missing the left antenna, otherwise the specimen is complete. The palpi in the female are broken, but still attached to the mouth parts. The female was originally thought to be a different species; however, the DNA match with the male of *Cicadomorphuschuya*.

**Figures 26–42.**
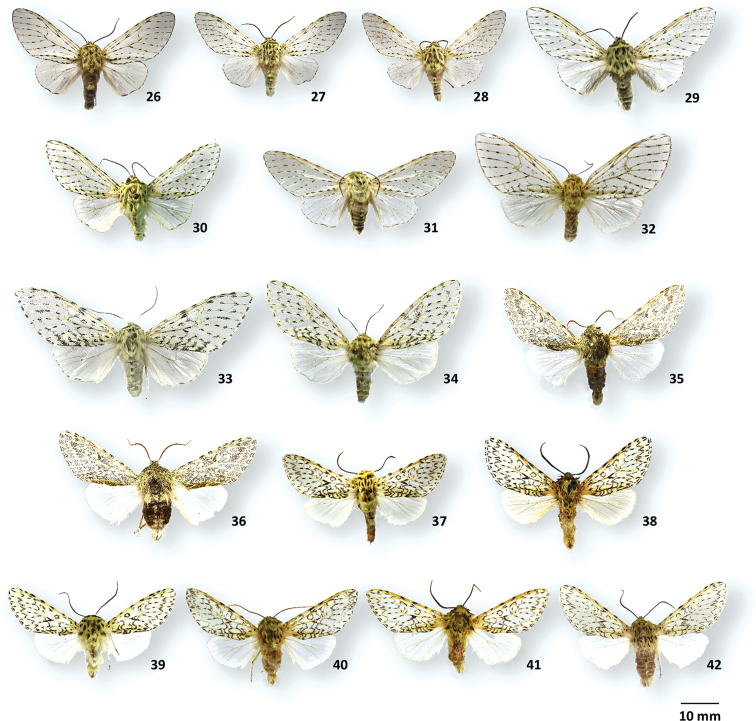
Adult habitus of *Gaujonia*, *Gaujoptera*, and *Millerana* species **26***Gaujoniaarbosi*, ♂, MGCL, Napo, Ecuador **27***G.bichu*, ♂, holotype, FSU, Zamora-Chinchipe, Ecuador **28***G.bichu*, ♂, paratype, FSU, Loja, Ecuador **29***G.chiqyaq*, ♂, paratype, MGCL, Marinio, Colombia **30***G.kanakusika*, ♂, holotype, MGCL, Cundinamarca, Colombia **31***G.sourakovi*, ♂, holotype, MGCL, Cusco, Peru **32***G.arbosi*, ♀, MGCL, Napo, Ecuador **33***G.chiqyaq*, ♀, holotype, MGCL, Marinio, Colombia **34***G.kanakusika*, ♀, paratype, MGCL, Cundinamarca, Colombia **35***Gaujopteraamsa*, ♂, holotype, MGCL, Cotapata, Bolivia **36***G.amsa*, ♂, paratype, MGCL, Junin, Peru **37***Milleranaarbosioides*, ♂, MGCL, Carchi, Ecuador **38***M.austini*, ♂, holotype, CUIC, Cotopaxi, Ecuador **39***M.matthewsae*, ♂, holotype, MGCL, Ancash, Peru **40***M.cundinamarquensis*, ♂, holotype, MGCL, Cundinamarca, Colombia **41***M.tigrina*, ♂, holotype, MGCL, Carchi, Ecuador **42***M.cajas*, ♂, holotype, MGCL, Azuay, Ecuador.

#### Cicadomorphus falkasiska sp. nov.

Taxon classificationAnimaliaLepidopteraNoctuidae

3AB0F17E-52EB-5263-B50F-9629138DC35E

http://zoobank.org/894304E1-B33B-44CC-9E71-F22AA0D09ED2

Figs 3 , 18 , 24 , 25 , 61 , 83 , 91A , 92

##### Type material.

***Holotype*** ♂, **Peru**: Peru, Oxapampa near Villa Rica, 2700 m, 21 Feb. 2018, coll. Falk Zahlaus / reared with *Quercusaquifolioides* & *Prunuslaurocerasus* / UF, FLMNH, MGCL 1049146. DNA voucher MGCL-NOC-65346 deposited in MGCL. ***Paratypes*** (4 ♂, 3 ♀, MGCL) : **Peru**: same collecting data as holotype (2 ♂, 1 ♀); Peru-Pasco 9 km on 310° from Yapi, H = 2470 m, 10°41,8'S, 75°35,4'W, 11–13.02.2011, leg. Viktor Sinyaev & Alexander Poleschuk (2 ♂); Peru-Junin near Calabaza vill., 11°30.4'S, 74°51.7'W, 20.12.2010, H = 2722 m, leg/coll. Viktor & Svetlana Sinyaev + Vladimir Izerskiy (2 ♀). **Additional examined specimens** (2 ♂, 1 ♀, TK): **Peru**: same collecting data as holotype.

##### Etymology.

*Falkasiska* is the combination of the names from Falk Zahlaus who collect the first female specimen and Toni Kasiske, who reared the eggs obtained from that female.

##### Diagnosis.

*Cicadomorphusfalkasiska* and *C.chicharra* share certain characters, especially in coloration and wing pattern. However, *C.falkasiska* is paler and the orbicular spot larger and outlined in black; also, the subterminal line is quite visible in *C.chicharra*, whereas in *C.falkasiska* it is inconspicuous. The male genitalia have a relatively wide aedeagus; the vesica is large and semi-rounded; the female genitalia have a wide appendix bursae and corpus bursae.

##### Description.

***Head*.** Third segment of palp in both sexes black with a pale-yellow stripe ventrally and a dot of same color dorsally; frons darker-yellow than rest of body; antenna dark brown. ***Thorax*.** Pale yellow with some black blurred spots, which are more visible in male than female. ***Wings*.** Forewing length: male 23–25 mm; female 30–32 mm; forewing pale yellow; hyaline areas in male nearly without scales, whereas forewing slightly more covered with scales in female; postmedial and subterminal lines inconspicuous in male, whereas basal, antemedial, and medial lines well defined; female with all lines weakly defined; orbicular spot large and elongate; reniform spot slightly wide, outlined with black scales and with a black dot in middle; hindwing hyaline in male, semi-hyaline in female with fringe paler yellow than forewing. ***Leg.*** Pale yellow with prothoracic legs with some brown patches. ***Abdomen*.** Whitish gray with some tufts over middle of abdomen; tufts on A1–A3 yellow with a small black dot on A2, whereas remainder tufts on other segments are black with some yellow and white scales; female with yellow tufts on A1–A4. ***Male genitalia*.** Cucullar region relatively wide with an axe-shaped lobe; apex round with a lobe and apex covered with quite long setae; saccular region wide with process of sacculus thin and curved; juxta flat on upper side but with a small V-shaped depression in middle; aedeagus relatively wide at opening to vesica; aedeagus ca. as long as vesica; vesica rounded with narrow transverse band of spines. ***Female genitalia*.** Anal papilla long and wide with posterior apophysis ca. as long as anal papilla; anterior apophysis short; sterigma large, fused above ostium; ductus bursae wide and short; and appendix bursae large, well sclerotized; corpus bursae not sclerotized 1¼ × longer than appendix bursae.

##### Immature stages.

***Egg.*** Pale yellow, turning dark brown close to emergence. ***Larva*.** Body black with yellowish orange verrucae and short setae; a set of long white setae on lateral verrucae, prothorax near head capsule, and on last tergite (Fig. 91A). ***Pre-pupa*.** Similar to last instar but with verrucae and setae dark yellow. *Pupa.* Dark brown.

##### Distribution.

All specimens were found in central Peru at a high altitude of ca. 2500 m or above (Fig. 92).

##### Biology.

*Cicadomorphusfalkasiska* is the only species in the genus for which immature stages are known. Adults are active throughout the year, but especially so in spring and summer. *Cicadomorphusfalkasiska* feeds on *Prunus* spp. especially on *P.subcorymbosa* Ruiz ex Koehne (JIM, pers. obs.).

##### Remarks.

Holotype (Fig. 18) and paratypes are well preserved, but three of the specimens were destroyed and only identifiable by genitalia dissection.

**Figures 43–58.**
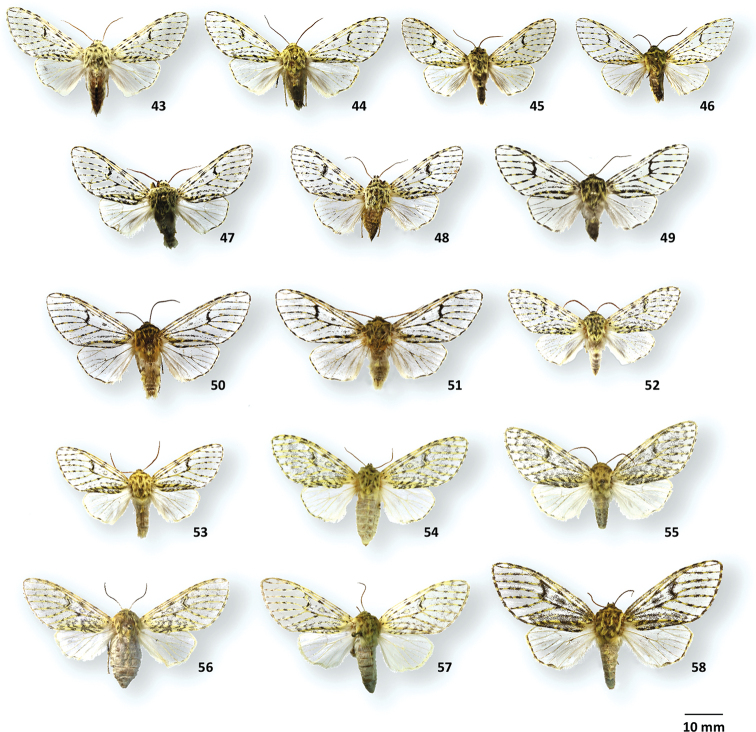
Adult habitus of *Oculicattus* species **43***Oculicattusrenifera*, ♂, MGCL, Cusco, Peru **44***O.renifera*, ♂, MGCL, Junin, Peru **45***O.schmidti*, ♂, holotype, MGCL, Pasco, Peru **46***O.schmidti*, ♂, paratype, MGCL, Junin, Peru **47***O.inca*, ♂, holotype, MGCL, Sierra Siberia, Bolivia **48***O.boliviana*, ♂, holotype, MGCL, Sierra Siberia, Bolivia **49***O.uturunku*, ♂, holotype, MGCL, Morona, Ecuador **50***O.raizae*, ♂, holotype, MGCL, Tolima, Colombia **51***O.raizae*, ♂, paratype, ZSM, Pichincha, Ecuador **52***O.brehmi*, ♂, holotype, FSU, Loja, Ecuador **53***O.brehmi*, ♂, paratype, MGCL, Napo, Ecuador **54***O.brehmi*, ♀, paratype, MGCL, Napo, Ecuador **55***O.brehmi*, ♀, paratype, MGCL, Napo, Ecuador **56***O.renifera*, ♀, MGCL, Cusco, Peru **57***O.renifera*, ♀, MGCL, Cusco, Peru **58***O.raizae*, ♀, paratype, MGCL, La Paz, Bolivia.

#### Cicadomorphus lilianae sp. nov.

Taxon classificationAnimaliaLepidopteraNoctuidae

0BA64973-144F-5487-9A31-62105E8A3C1E

http://zoobank.org/0B2743EF-09FA-4AB2-9850-39DFB1D3C5C4

Figs 16 , 22 , 60 , 82 , 92,

##### Type material.

***Holotype***: ♂, **Ecuador**: Ecuador, Napo, Cosanga, 2150 m, 29 Mar. 1976, coll. N. Venedictoff Holotype ♂. Deposited in MGCL. ***Paratypes*** (8 ♂, 3 ♀, MGCL): **Ecuador**: Ecuador, Napo + 10km Papallacta, 2730 m, 13–15 Sep. 1982, coll. N. Venedictoff (7 ♂); Ecuador: Napo, El Carmelo Barbar’ 10 km, 2750 m, 19 Jan. 1985, coll. N. Venedictoff (1 ♀). (1 ♂, CNC): **Ecuador**: Ecuador, Napo province, 23 km road La Bonita, 2400 m, 7–9 Apr. 1986, coll. Stuart McKamey. (1 ♀, FSU): **Ecuador**: Ecuador, Zamora-Chinchipe Parque Nacional Podocarpus ridge forest, Bombuscaro area, Blacklight 2 × 15W (40), 04°06.84'S, 78°57.97'W, 23.iii. 2011, 19.30–20.30 h, ca. 1120 m, Gunnar Brehm leg. / DNA Barcode run 2011, COI-5P marker, University of Guelph / Arcec 32423 / [Arcec 32284] (1 ♀). **Additional examined specimens** (2 ♂ UNAB): **Colombia**: Colombia, Cundinamarca, Guasca, Choachi, Vda. El Curi, F. panes de Roka 1996 m, 02 Jun. 2013, coll. V. Raigozo (2 ♂).

##### Etymology.

This species is named in honor of my sister Lilian Martinez Canto (1989–2017) for her love, charisma, and support offered during all her beautiful life.

##### Diagnosis.

*Cicadomorphuslilianae* is small and the wing pattern is blurred, causing the lines to appear shapeless. The males have darker coloration than the females, and the line pattern is better defined. This species shares some features in genitalia with *C.chuya*, such as the tapered vesica.

##### Description.

***Head*.** Palp black, terminal segment admixed with brown and white scales; frons dark yellow basally with some black scales; female ground color pale yellow with gray scales; antenna brownish orange. ***Thorax*.** Ground color dark yellow with some black tufts; female same color but with gray tufts; collar with black ground color with margins yellow, female with ground color gray. ***Wings*.** Both sexes dark yellow, similar in pattern, nevertheless female is paler, and wing pattern is basally almost imperceptible; forewing length: male 25–27 mm; female 31–33 mm; forewing yellow with pattern in black, but blurrier, male darker; both sexes with semi-hyaline areas with some scattered dark yellow scales; both sexes with blurry lines; orbicular spot elongate, ca. same size in both sexes; reniform spot heavily outlined with black scales; hindwing hyaline with fringe yellow, but gray on posterior margin. ***Legs*.** Yellow, except prothoracic legs, which are brown with some black scales. ***Abdomen*.** Gray with yellow tufts in middle of abdomen and on each side; brown tufts on A3–A7; female abdomen yellow with some brown scales. ***Male genitalia*.** Cucullus wide with apex wide; costal margin without setae; outer margin sharply bent; tooth-like protuberance small; lobe with external apex large; posterior margin of lobe curved; sacculus wide with narrow process touching tooth-like protuberance; saccus thin; tegumen narrow, lightly sclerotized, barely visible around uncus; juxta wide, upper side flat; aedeagus relatively wide, V-shaped in opening; tapered vesica; band of spines with a rounded ending. ***Female genitalia*.** Anal papilla small; relatively wide posterior apophysis 1 ⅓ × longer than anal papilla; sterigma open trapezoid shaped; anterior apophysis short; ductus bursae small, well-sclerotized; appendix bursae broad, sclerotized; corpus bursae ⅓ × longer than appendix bursae.

##### Immature stages.

Unknown.

##### Distribution.

This species has been found in Ecuador and Colombia at very variable elevations from 1100–2700 m (Fig. 92).

##### Biology.

Unknown.

##### Remarks.

The tip of the left antenna from the type specimen was broken during examination (Fig. 16). The paratype female is intact; however, the left orbicular spot is incomplete (Fig. 22). Otherwise, the female forewing is more brightly colored than that of the male basally where the antemedial and basal lines are almost inconspicuous. The female paratype from FSU has a different DNA voucher label from the voucher published at http://barcodinglife.com.

**Figures 59–63.**
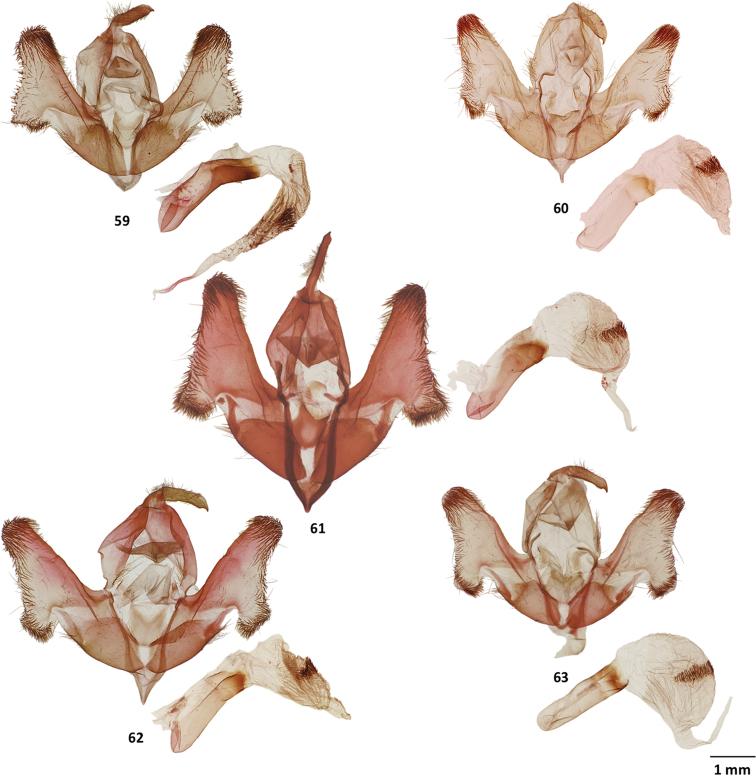
Male genitalia of *Cicadoforma* and *Cicadomorphus* species **59***Cicadoformaocelotus*, holotype, MGCL, Santander, Colombia **60***Cicadomorphuschuya*, holotype, MGCL, Tolima, Colombia **61***C.falkasiska*, holotype, MGCL, Oxapampa, Peru **62***C.lilianae*, holotype, MGCL, Napo, Ecuador **63***C.chicharra*, holotype, MGCL, San Rosa, Bolivia.

#### Gaujonia

Taxon classificationAnimaliaLepidopteraNoctuidae

Dognin

04D4F827-BEFA-5B00-8074-585E7A984459

##### Gender.

Masculine.

##### Type species.

*Gaujoniaarbosi* Dognin, 1891. Descriptions de Lépidoptéres nouveaux. Le Naturaliste, 13: 126.

##### Etymology.

Paul Dognin probably named this genus Gaujonia after Fr. Theophile Gaujon a Catholic Lazarist missionary priest and entomologist residing in Loja, Ecuador, who helped by collecting specimens for him.

##### Included species.

Within the genus *Gaujonia* there were previously four species, however, three of the species belong to three different genera: *Gaujoniaarbosioides* Dognin (*Milleranaarbosioides* (Dognin), comb. nov.), *G.renifera* Hampson (*Oculicattusrenifera* (Hampson), comb. nov.), and *G.vau-nigrum* Hampson (*Cicadoformavau-nigrum* (Hampson), comb. nov.) based on genetic and morphological differences. Only one of the original species remains in *Gaujonia*, it being the type species *Gaujoniaarbosi* Dognin. However, during this revision four new species were discovered: *Gaujoniabichu* sp. nov., *Gaujoniachiqyaq* sp. nov., *Gaujoniakanakusika* sp. nov., and *Gaujoniasourakovi* sp. nov.

##### Diagnosis.

*Gaujonia* is similar to *Oculicattus* morphologically, but it is most closely aligned genetically with *Cicadoforma* and *Cicadomorphus* (Fig. 1). The interfacetal setae of *Gaujonia* are longer than those in *Cicadoforma* and *Cicadomorphus*. Male genitalia are similar to those of *Oculicattus*, however, the cucullus is wider and slightly square in shape, whereas in *Oculicattus* the cucullus is thinner and rounded. *Gaujonia* has a short vesica and two patches of spines, whereas in *Oculicattus* the vesica is longer with three patches of spines. Female genitalia of *Gaujonia* are similar to that of *Oculicattus*, but that of *Oculicattus* is larger in size, however, the sterigma is smaller in that genus than in *Gaujonia*. DNA barcoding corroborated that *Gaujonia* is closer to *Cicadoforma* and *Cicadomorphus* (~ 6% divergent) than *Oculicattus* (~ 8%).

##### Description.

Sexually dimorphic in size, female larger than males; forewing and hindwing hyaline with scales only on wing veins; orbicular and reniform spots in male inconspicuous or absent, whereas female has an orbicular spot and narrow reniform spot. Antenna filiform and long haustellum dark brown; eyes hairy with long interfacetal setae. Male genitalia with saccular and cucullar regions separated and clasper absent; short-beaked uncus; aedeagus wide and vesica short with two sclerotized patches of spines on each side. Female genitalia with sterigma open wide, ductus bursae and appendix bursae are heavily sclerotized, whereas corpus bursae is not.

##### Immature stages.

***Egg.*** Circular, slightly flattened; chorion forming large square cells making the surface slightly rugose. ***Larva*.** Undergo from five to seven instars. Late instars with dense bands of secondary setae on the abdominal tergites, which are more scattered on the thorax; verrucae have scattered long setae; two large prothoracic verrucae with long tufts of secondary setae ***Pupa*.** Similar in features to other genera in the *Gaujonia* genus group (see *Cicadomorphus* diagnosis).

##### Biology.

The biology is known only from one species in *Gaujonia* (see *G.kanakusika* immature stages) (JIM, pers. obs.).

### Key to species of the genus of *Gaujonia* based on adult male morphology

**Table:** 

1	Vesica presenting short patches of spines (Figs 64–66)	**2**
–	Vesica showing elongated patches of spines giving the appearance of long hairs (Fig. 67)	***G.sourakovi***
2	Valva with apex flattened (Figs 64, 65)	**3**
–	Valva with apex rounded (Figs 8, 66)	**4**
3	Forewing with V-shaped mark on base of CuA2; cucullar region widely open, almost touching saccular region (Figs 29, 65)	***G.chiqyaq***
–	Forewing without V-shaped mark on base of CuA2; cucullar and saccular areas remarkably narrow (Figs 27, 28, 64)	***G.bichu***
4	Hindwing with fringe completely yellow with some small lines in black; cucullar region wide; vesica with two patches of spines similar in size (Figs 30, 66)	***G.kanakusika***
–	Hindwing with black fringe and minute spots at the end of each vein; cucullar region narrow; vesica with two patches different in size being small patch ½ × smaller than the other patch. (Figs 8, 26)	***G.arbosi***

#### Gaujonia arbosi

Taxon classificationAnimaliaLepidopteraNoctuidae

Dognin

A20BC6C5-4A35-5DBC-AFFA-8B36E804FB06

Figs 8 , 26 , 32 , 85 , 93

Gaujonia arbosi Dognin, 1891: 126.

##### Type material.

***Lectotype*** ♂, **Ecuador**: “*Gaujonia arbosi* type ♂ Dgn. / Environs de Loja Equateur / 1993 / Type No. 30907 U.S.N.M. / Genitalia slide m, Franclemont USNM 33564”, coll. P. Dognin ♂. Deposited in USNM. **Additional examined specimens** (4 ♂, 2 ♀, MGCL): **Ecuador**: Ecuador, Napo + 10km Papallacta, 2730 m, 13–15 Sep. 1982, coll. N. Venedictoff (3 ♂); Ecuador, Napo, El Carmelo Barbar’ 10km, 2750 m, 19 Jan. 1985, coll. N. Venedictoff (2 ♀). **Colombia**: Colombia, Putumayo near San Francisco 01°07'36"N, 076°50'30"W, 25–27.01.2018, 2525m., Leg Viktor Sinjaev and Juan Machado / (1 ♂).

##### Etymology.

Paul Dognin probably named this species *arbosi* after Fr. Mariano Arbós, a friend of Fr. Theophile Gaujon.

##### Diagnosis.

*Gaujoniaarbosi* is similar to *G.chiqyaq*, but it can be identified by coloration, which is dark yellow in *G.arbosi*. Both sexes are similar with the only difference being that the female is covered with brighter yellow scales from the fold to the posterior margin of the forewing, accenting the lines. For males the forewing length ranges from 17–19 mm and for females from 23–25 mm. Palp short and black; antenna has a stripe of dark yellow scales from the base to the seventh antennomere; antennae are black, and longer than in *G.chiqyaq*. Male thorax dark yellow with some patches of black. Forewing with pattern similar to that of *G.chiqyaq*, but the lines on Sc+R1 and posterior margin are thicker; there is a small dot in the middle of the base of the cell M1, which is not present in *G.chiqyaq*; the V-shaped mark at the base of CuA2 smaller and thicker than in *G.chiqyaq*. The hindwing, base of M2 is angled diagonally forward to the base of the wing, fused to the base of CuA1; in *G.chiqyaq* M2 is squared and not fused with the base of CuA1; the fringe is black with few yellow scales making minute spots at the end of the veins, except the cell A2, which is completely outlined in black. The male genitalia have the cucullar region wide, slightly diagonal to the base, whereas the cucullar region in *G.chiqyaq* is wide, opened, almost touching the saccular region; the juxta is semicircular in shape; the saccus is short and wide. Female genitalia, the sterigma is peanut shaped, and the corpus bursae is ⅛ × larger than the appendix bursae. DNA Barcoding showed that *G.arbosi* and *G.chiqyaq* are sister species with ca. ~ 0.3% of difference, but the morphological characters mentioned above distinguish both species.

##### Immature stages.

***Egg.*** Bright green and turning dark brown close to emergence. ***Larva*.** Only known from the third instar, which is very similar to that of *G.kanakusika*, but the body of *G.arbosi* is white with dark brown secondary setae, whereas the body of *G.kanakusika* is rather whitish yellow. ***Pre-pupa*.** Unknown. ***Pupa*.** Dark brown (Guevara and Romero 2008).

##### Distribution.

This species is endemic to coniferous and deciduous forest of Ecuador and Colombia and can be found at high elevations above 2500 m (Fig. 93).

##### Biology.

Guevara and Romero (2008) were the first to record the larval stages in *Gaujoniaarbosi* feeding on *Alnusacuminata* Kunth but they misidentified it as *Gaujoniaarbosioides* (now *Milleranaarbosioides*), even though the immature stages differ greatly between both genera (see *Millerana* immature stages). Additionally, the illustrations from Guevara and Romero (2008) lack of good quality to use for identification.

##### Remarks.

The holotype of the species was “missing” in the P. Dognin collection (USNM), which was a female illustrated by Dognin (1894). A male was listed as the type specimen, but this is actually a different species from the illustration made by P. Dognin (Fig. 8). Presumably both specimens were used in the description of the species in his second work Dognin (1894), and is also deposited in the Dognin collection (USNM), I designated the male as the lectotype for *Gaujoniaarbosi* Dognin under the provisions of Article 74.1.1 of the International Code of Zoological Nomenclature (1999), whereas the female is considered a different species (see *Gaujoniachiqyaq* remarks).

**Figures 64–68.**
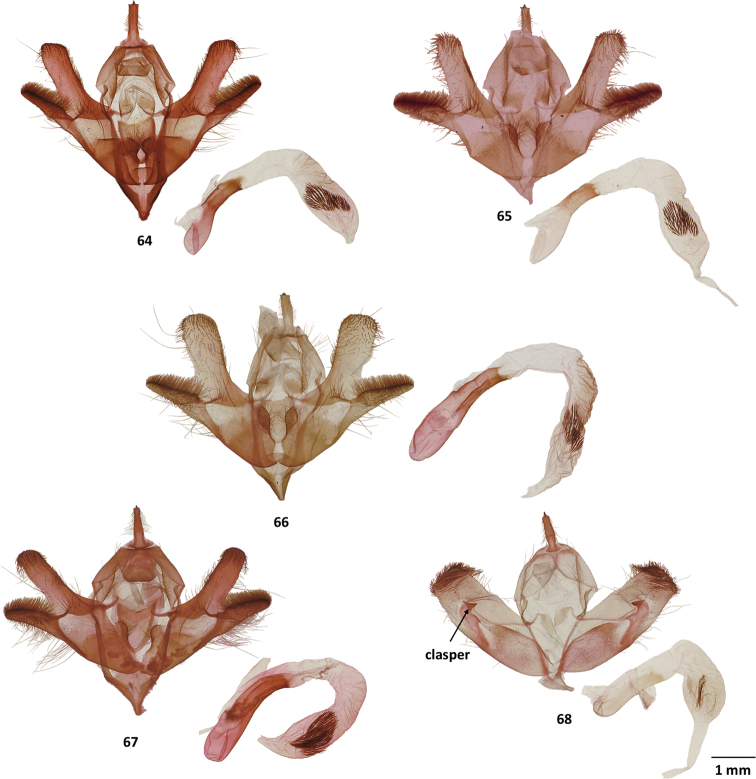
Male genitalia of *Gaujonia* and *Gaujoptera* species **64***Gaujoniabichu*, holotype, FSU, Zamora-Chinchipe, Ecuador **65***G.chiqyaq*, paratype, MGCL, Marinio, Colombia **66***G.kanakusika*, holotype, MGCL, Cundinamarca, Colombia **67***G.sourakovi*, holotype, MGCL, Cusco, Peru **68***Gaujopteraamsa*, holotype, MGCL, Cotapata, Bolivia.

#### Gaujonia bichu sp. nov.

Taxon classificationAnimaliaLepidopteraNoctuidae

F46C233F-0A1C-59B3-B6BF-BDE35919E693

http://zoobank.org/D18A59B3-47DA-4583-9274-D4376ADE594F

Figs 27 , 28 , 64 , 93

##### Type material.

***Holotype*** ♂, **Ecuador**: Ecuador, Zamora-Chinchipe ca 2 km E pass El Tiro rainforest, 30 m N of road, Blacklight 2 × 15W (61), 03°59.46'S, 79°07.58'W, 3.iv.2011, | 18.45–19.45, 2630 m, coll. Lisa Lehner / DNA Barcode run 2011, COI-5P marker, University of Guelph / Arcec 31087. [DNA voucher Arcec 31087] deposited in FSU. ***Paratypes*** (2 ♂, FSU): **Ecuador**: Ecuador, 8 km SE of Loja, Parque Nacional Podocarpus Cajanuma, mont. Rainforest, Blacklight 2 × 15W (50), 04°06.86'S, 79°10.48'W, 20.ix.2008, 2897 m, Florian Bodner leg / Arcec 32151 / Barcoding failed.

##### Etymology.

The term *bichu* is used to refer to an insect or small animal in the Quechua language. Since this is the smallest species of *Gaujonia*, this name seems appropriate.

##### Diagnosis.

*Gaujoniabichu* is closely related to *G.sourakovi*, but *G.bichu* is the smaller. The male genitalia have the valva and the saccular region narrow, and the process of the sacculus is longer and thinner.

##### Description.

***Head*.** Palp black; frons yellow with some black scales. ***Thorax*.** Dorsally covered by large black patches. ***Wings*.** Male yellow with thin spots and lines on venation; forewing length: male 15–17 mm; forewing with two V-shaped marks on base of R1+R2 and R4+R5; base of cell with small black dot on M1; five black dots on Sc+R1 with a line right on apex; half of posterior margin black; hindwing with fringe black, interrupted by a yellow dot at end of each vein. ***Leg.*** Prothoracic leg black with femur and tarsi presenting yellow spots; mesothoracic legs yellow with some black spots; metathoracic legs yellow. ***Abdomen*.** Grayish yellow with yellow and black tufts in middle of abdomen dorsally on A1–A5, remainder of abdomen covered in yellow scales. ***Male genitalia*.** Valva long and narrow a little swollen from middle to apex externally; sacculus narrow; sacculus process long and narrow; juxta shield-like; tegumen wide, narrower near valva; aedeagus short and narrow; vesica elongated with two oval patches, one 2 × longer than the other.

##### Immature stages.

Unknown.

##### Distribution.

*Gaujoniabichu* specimens were found in Ecuador in coniferous and deciduous forests at high elevations above 2500 m (Fig. 93).

##### Biology.

Unknown.

##### Remarks.

Three specimens are in good condition; one specimen from Loja has no black dots on the base of cell M1.

#### Gaujonia chiqyaq sp. nov.

Taxon classificationAnimaliaLepidopteraNoctuidae

6DCEF0E9-53A5-508B-A134-4103BF5EE01F

http://zoobank.org/38C22D4A-F2D3-4ED4-9A71-578970C72AF5

Figs 29 , 33 , 65 , 86 , 93

##### Type material.

***Holotype*** ♀, **Colombia**: Colombia, Putumayo, Municipio San Francisco, Antenas on Bosque Siberia, 01°08'45"N, 76°50'43"W, 29–30.01.2018, 2940 m., Leg Viktor Sinjaev and Juan Machado / UF, FLMNH, MGCL 1049015. [DNA voucher MGCL-NOC-65199] deposited in MGCL. ***Paratypes*** (7 ♂, 1 ♀, MGCL): **Colombia**: Colombia, Marinio Laguna, the Cocha El Carrizo, 01°10'35"N, 77°08'13"W 25.01.2018, 2900 m., Leg Viktor Sinjaev and Juan Machado / UF, FLMNH, MGCL 1049007, [DNA voucher MGCL-NOC-65191] (1 ♂); : Colombia, Marinio Laguna, the Cocha near Encano, 01°08'10"N, 77°11'07"W, 21–24.01.2018, 2900 m., Leg Viktor Sinjaev and Juan Machado (1 ♂); : Colombia, Boyacá, Vereda El Palmar, Parque Nacional El Virolín, 6°02'28’’N, 73°13'18’’W, 2114m, 25.11.2013, legit Victor Sinyaev & Mildred Márquez (2 ♂); Colombia, Border Narinio-Putumao near Pasto, 01°08'52"N, 77°05'59"W, 17–19.01.2018, 3200 m., Leg Viktor Sinjaev and Juan Machado (1 ♀); Ecuador, Pichincha, Camping Bella Vista, 2230 m, 0°00'41"S, 78°41'17"W, 19. XII 2012–7. I 2013, leg. Sinjaev & Romanov & [coll.] Dr. R. Brechlin (1 ♂); **Peru**: Peru-Junin Near Calabaza vill., 11°29,8'S, 74°51,9'W, 17.12.2010, H = 2964 m, leg/coll. Viktor & Svetlana Sinyaev + Vladimir Izerskiy (1 ♂); Peru-Junin near Calabaza vil., 11°30.5'S, 74°49.4'W, 1–2.02.2011, H = 2137 m, leg. Viktor Sinyaev & Alexander Poleschuk / UF, FLMNH, MGCL 1049018. [DNA voucher MGCL-NOC-65202] (1 ♂).

##### Etymology.

The name *chiqyaq* means green in the Quechua language, referencing the diagnostic green coloration of this species.

##### Diagnosis.

Beside the features mentioned above to differentiate *Gaujoniachiqyaq* from *G.arbosi*, there are also other informative characters to identify this species. The male has a narrow reniform spot outlined by black scales along the upper and lower sides, but not in the middle region. The orbicular spot is small and greenish yellow, outlined in black. The thorax has large black tufts. The female has a larger reniform spot with the same yellow coloration. Forewing transverse lines are more sharply defined and thicker than in *G.arbosi*.

##### Description.

***Head*.** Palp with last segment black; frons with a combination of black and greenish yellow scales. ***Thorax*.** Covered with long black tufts. ***Wings*.** Both sexes greenish yellow with thin spots and lines; forewing length: male: 20–22 mm; female: 28–30 mm; forewing ground color greenish yellow with small, thin lines on venation forming pattern, female with thicker and well-defined lines; orbicular spot in male small, black, but almost imperceptible; orbicular spot larger in female and elongated; reniform spot in both sexes narrow, greenish yellow, outlined in black; both sexes with hindwing venation green, and fringe black with some greenish yellow dots. ***Leg.*** Metathoracic and metathoracic legs black with some greenish yellow patches; hind legs greenish yellow with black shading. ***Abdomen*.** Brown dorsally with green tufts on A1–A3 and some black scales in middle of the green tufts; ventrally greenish yellow; female similar to male but paler in color. ***Male genitalia*.** Cucullar region extended close to saccular region, valva relatively narrow, slightly ridged externally on apex; saccular region rounded at base and process wide; saccus wide V-shaped; juxta rectangular concave on top; tegumen wide; aedeagus short 3 × longer than wide; basal area almost same size as apical part of vesica with two patches of spines, one 2 × narrower than the other. ***Female genitalia*.** Anal papilla wide, slightly ridged; posterior apophysis ⅓ × longer than anal papilla; sterigma large oval shaped, fused above ostium; anterior apophysis short; ductus bursae heavily sclerotized and connected to appendix bursae, which is sclerotized as well; appendix bursae ½ × narrower than corpus bursae.

##### Immature stages.

Unknown.

##### Distribution.

This species has a broad distribution from Ecuador to Peru between 2100– 3200 m (Fig. 93).

##### Biology.

Unknown.

##### Remarks.

The holotype of *Gaujoniachiqyaq* closely resembles the type specimen of *Gaujoniaarbosi* (Fig. 33) illustrated by Dognin (1894). However, it is a different species from the lectotype of *G.arbosi*, differing from it in characters of the male and female genitalia, thus it is here established as a new species. The holotype of *Gaujoniaarbosi* and most of the paratypes are well preserved. Guevara and Romero (2008) misidentified this species as *Gaujoniaarbosioides*, which is placed in a different genus (see *Milleranaarbosioides* remarks).

#### Gaujonia kanakusika sp. nov.

Taxon classificationAnimaliaLepidopteraNoctuidae

F5BE7255-BB81-583E-BF56-60D37140F969

http://zoobank.org/AE542D1A-A612-4F37-922D-F48482F5D6F7

Figs 4 , 30 , 34 , 66 , 87 , 91B , 93

##### Type material.

***Holotype*** ♂, **Colombia**: Colombia, Cundinamarca, Guasca, El Chochal de Siecha, 3120 m, 28 Nov. 2019, coll. Jose I. Martinez / UF, FLMNH, MGCL 1049048. [DNA voucher MGCL-NOC-65232] deposited in **MGCL**. ***Paratypes*** (7 ♂, 2 ♀, **MGCL**): **Colombia**: Same collecting data as holotype (1 ♂); Colombia, Cundinamarca dpt, Vereda La Concepción, Bosque La Guajira, 4°47'34’’N, 75°46'60’’W, 8–12.XI.2014, 2910 m, leg. V. Sinyaev & M. Márquez (2 ♂); Colombia, Cundinamarca, Municipio Guasca, Alto El Paramo, 04°53'44"N, 73°45'46"W, 29–31.12.2017, 3250 m., Leg Viktor Sinjaev and Juan Machado (1 ♂); Colombia, Cundinamarca, Municipio Guasca, near Alto El Paramo, 04°53'55"N, 73°46'21"W, 22–23.02.2018, 3070 m. Leg Viktor Sinjaev and Juan Machado (3 ♂, 2 ♀)

##### Etymology.

The name is formed from the Quechua words *kanaku* (fire) and *sika* (caterpillar), based on the immature stages of this species (Fig. 91B).

##### Diagnosis.

This species can be differentiated from other species by the sulfur-yellow coloration of most of the body, including the abdomen.

##### Description.

***Head*.** Palp with basal and second palpal segment sulfur-yellow, last segment with white tip; frons with small black line; female with similar wing pattern, but brighter in coloration. ***Thorax*.** Sulfur-yellow, covered with small black dots. ***Wings*.** Forewing sulfur-yellow; length: male 18–20 mm; female 27–29 mm; forewing with lines visible from posterior margin to fold; basal and antemedial lines almost completely developed; no presence of a V-shaped mark; Hind wing fringe same yellow color as body. ***Legs*.** Prothoracic and mesothoracic legs black with some sulfur-yellow spots on joints; tarsal scales yellow and black. ***Abdomen*.** Sulfur-yellow with a small area of gray in middle of dorsum divided by black and yellow tufts. ***Male genitalia*.** Cucullus large, rounded apically; sacculus narrow with a long process; juxta shell-shaped with apices pointed; tegumen wide; saccus narrow; aedeagus short and wide; vesica 1 ½ x as long as aedeagus with two large spine patches ca. same size. ***Female genitalia***. Anal papilla small, semicircular; posterior apophysis ⅓ × longer than anal papilla; sterigma narrow and large, semi-rectangular; anterior apophysis relatively long and heavily sclerotized; corpus bursae 1 ½ × longer than appendix bursae.

##### Immature stages.

***Egg.*** Completely light green, turning dark brown close to emergence. ***Larva*.** In general, body pale orange with dark pink verrucae and neon-orange setae on dorsal verrucae; long pale-yellow setae on lateral verrucae; inter-tergal membrane brown with white dots (Fig. 91B). ***Pre-pupa*.** Darker than ultimate instar. ***Pupa*.** Dark brown with white setae covering the whole body.

##### Distribution.

All specimens were found in deciduous forests from the central-north Colombia between 2900– 3300 m (Fig. 93).

##### Biology.

*Gaujoniakanakusika* is the second species in this genus known to feed on *Alnusacuminata* Kunth. Adults fly year-round, mainly in summer (JIM, pers. obs.).

##### Remarks.

Holotype (Fig. 30) and paratypes in good condition. This species was the only one whose phylogenetic placement had low support in the gene tree; however, it shares apomorphic characters with other *Gaujonia* species, so it is maintained as a *Gaujonia*.

**Figures 69–73.**
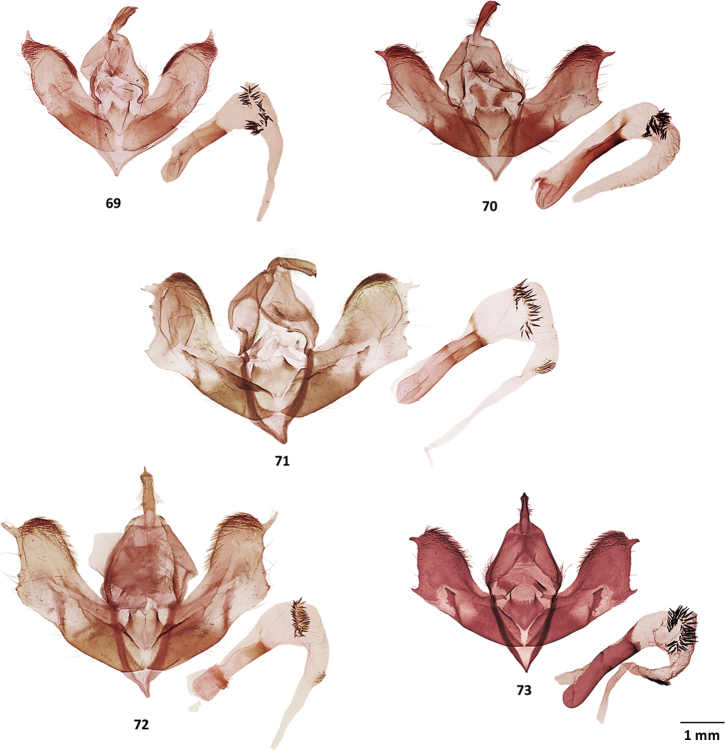
Male genitalia of *Millerana* species **69***Milleranacundinamarquensis*, holotype, MGCL, Cundinamarca, Colombia **70***M.tigrina*, holotype, MGCL, Carchi, Ecuador **71***M.matthewsae*, holotype, MGCL, Ancash, Peru **72***M.cajas*, holotype, MGCL, Azuay, Ecuador **73***M.austini*, holotype, CUIC, Cotopaxi, Ecuador.

#### Gaujonia sourakovi sp. nov.

Taxon classificationAnimaliaLepidopteraNoctuidae

D7E01EB2-1501-5EAC-93E9-A8D818451487

http://zoobank.org/5ADB338A-B431-4B31-8A9E-7518B2B02939

Figs 31 , 67 , 93

##### Type material.

***Holotype*** ♂, **Peru**: Peru, Department Cuzco, Manu Park, San Pedro, 1800 m, Mar. 1997, coll. local people. Deposited in MGCL. **Additional examined specimens** (1 ♂, MGCL): **Peru**: Same collecting data as holotype.

##### Etymology.

This species was found by my colleague and friend Andrei Sourakov when he was helping me locate additional specimens deposited in the MGCL, and thus I named it in his honor for his wonderful help.

##### Diagnosis.

*Gaujoniasourakovi* differs from other species mainly in coloration, having the most lightly marked forewing in this group. The male genitalia have the apex of the cucullus rounded, the saccular region is wide, and two patches of long hair-like spines on the vesica, differing from the others which have wider and well-developed spines.

##### Description.

***Head*.** Last segment of palp black with some yellow scales; frons yellow, shaded with black scales. ***Thorax*.** Dark yellow with black dorsally. ***Wings*.** Forewing length: male 19–21 mm; forewing scales only on venation and margins; V-shaped mark at base of CuA2 widely open; dot in the middle of base of cell M1 elongated; orbicular spot small; veins lined with black; hindwing fringe black with dark yellow scales at end of each vein, whereas area on posterior margin of hindwing brown; a black line at base of vein M3. ***Leg.*** Prothoracic and mesothoracic legs dark brown with yellow scales on joints, whereas metathoracic legs dark yellow with some dark brown spots. ***Abdomen*.** Brown with scattered black scales dorsally, paler ventrally; long yellow scales laterally; black tufts with some yellow and brown scales on A1–A4. ***Male genitalia*.** Cucullar area narrow and curved with apex rounded; saccular region and process wide; juxta flat on upper side; tegumen wide; aedeagus 2 ⅔ × longer than wide; basal area of vesica 4 × longer than wide and vesica almost same width with two large patches of hair-like spines, one ovoid.

##### Immature stages.

Unknown.

##### Distribution.

The species *Gaujoniasourakovi* was collected in a deciduous forest at 1800 m elevation in southeastern Peru (Fig. 93).

##### Biology.

Unknown.

##### Remarks.

One specimen has the left forewing broken at the base and a brown patch on cell R5. Right wing with some brown patches near the outer margin between the cells R5-M2. In the other specimen the wings and left valva are broken, so it is not included as a paratype.

#### Gaujoptera gen. nov.

Taxon classificationAnimaliaLepidopteraNoctuidae

7477128C-B284-571C-A57C-6ED98C41D09A

http://zoobank.org/5EBE13A2-1698-415E-AAFD-7AFEF8916772

##### Gender.

Feminine.

##### Type species.

*Gaujopteraamsa* sp. nov.

##### Etymology.

*Gaujoptera* is derived from the similarities with the genera *Gaujonia* and *Lichnoptera*.

##### Included species.

*Gaujoptera* is monotypic, the only species is *Gaujopteraamsa* sp. nov., which was misidentified as a *Gaujonia* species; however, morphological and molecular analyses resulted in recognizing a new genus that is positioned close to the genus *Millerana* (Fig. 1).

##### Diagnosis.

*Gaujoptera* is only known from two well-preserved male specimens and it has a close relationship with *Millerana*, not only genetically, but also by distinct morphological characters. Nevertheless, the wing pattern is more defined in *Millerana* than in *Gaujoptera*, which has a blurry pattern that is difficult to discern. In addition, the thorax is marbled with black, gray, brown, and sulfur-yellow scales, differing from species of *Gaujonia*, which have the black spots and patches well defined on the thorax. *Gaujoptera* does not have a V-shaped mark at the base of CuA2. The most important feature to differentiate *Gaujoptera* from *Gaujonia* is the presence of small clasper on the valva, similar to that of *Lichnoptera*.

##### Description.

Forewing and hindwing dark sulfur-yellow with an inconspicuous pattern, only reniform spot visible. Hindwing hyaline with scales only on margins. Antenna filiform, with a short brownish orange haustellum; eye hairy with short interfacial setae. Male genitalia simple in male with small triangular clasper; small, curved uncus; aedeagus shorter than vesica; vesica with small spines in middle. Female unknown.

##### Immature stages.

Unknown.

##### Biology.

Unknown.

#### Gaujoptera amsa sp. nov.

Taxon classificationAnimaliaLepidopteraNoctuidae

3E47E8A8-2A80-5EBC-902E-B2FF45A8BD1D

http://zoobank.org/EAE3EDDC-BED8-4CE2-8341-126A89FBAC5D

Figs 5 , 35 , 36 , 68 , 93

##### Type material.

***Holotype*** ♂, **Bolivia**: Bolivia, Cotapata, 16°16.8'S, 67°52.6'W, 6–7.1.2010, H = 3210 m, leg/coll. Viktor & Svetlana Sinyaev + Alexei Zamesov / UF, FLMNH, MGCL 1049124. [DNA voucher MGCL-NOC-65307] deposited in MGCL. ***Paratype*** (1 ♂, MGCL): **Peru**: Peru-Junin near Calabaza vill., 11°29,8'S, 74°51,9'W, 17.12.2010, H = 2964 m, leg/coll. Viktor & Svetlana Sinyaev + Vladimir Izerskiy (1 ♂). **Additional examined specimens** (6 ♂, MGCL): **Bolivia**: same collecting data as holotype. (3 ♂); **Peru**: same collecting data as paratype. (2 ♂); Peru: Dept. Junin, Cerro Pichita, Res. Sta. nr. San Ramon, 2965 m, 7–9 Apr. 2011, coll. J. B. Heppner & C. Carrera (1 ♂).

##### Etymology.

*Amsa* is a Quechuan word that means dark, opaque, or confused.

##### Diagnosis.

The species *Gaujopteraamsa* has similar morphological characters with species of *Millerana*, however, it is easy to identify because the thorax has no spots or patches, but is completely marbled with black, gray, brown, and sulfur-yellow scales. The forewing is dark sulfur-yellow with a blurry pattern in gray. Additionally, the genitalia have a simple valva without any lobe or protuberances, and a small clasper is present.

##### Description.

***Head*.** Palp with last segment black with few yellow scales on tip; frons marbled. ***Thorax*.** Marbled in black, gray, brown, and sulfur-yellow. ***Wings*.** Forewing length: male 18–20 mm; forewing marbled with wing pattern in gray; pattern blurry; orbicular spot barely visible in yellow; a narrow lunate marking on reniform spot; terminal lines zigzag; hindwing hyaline with sulfur-yellow fringe. ***Leg.*** Prothoracic and mesothoracic legs sulfur-yellow with some brown spots; tarsi brown and yellow; metathoracic legs marbled in brown and yellow with tarsi sulfur-yellow. ***Abdomen*.** Sulfur-yellow with a black line of tufts in middle of abdomen on A1–A5. ***Male genitalia*.** Valva rectangular, simple; saccular region wide; apex densely covered by setae; clasper small, triangular; juxta shell-like; tegumen narrow; uncus small; aedeagus short, almost same width as vesica; vesica ovoid with a line of minute spines in middle, positioned diagonally.

##### Immature stages.

Unknown.

##### Distribution.

*Gaujopteraamsa* occurs from central Peru to northern Bolivia at high elevations ca. 3000 m or above (Fig. 93).

##### Biology.

Unknown.

##### Remarks.

Most of the specimens are very damaged and could only be identified by genitalia examination, except the holotype and paratype (Figs 35, 36).

#### Millerana gen. nov.

Taxon classificationAnimaliaLepidopteraNoctuidae

F87312EB-4A73-5280-A96C-39711D7AE0D9

http://zoobank.org/F7EC8D53-E82F-4ECB-8896-210255E06389

##### Gender.

Feminine.

##### Type species.

*Gaujoniaarbosioides* Dognin, 1894. Lépidoptères de Loja et environs (équateur), descriptions d’espèces nouvelles 17: 87.

##### Etymology.

*Millerana* is dedicated to my mentor and friend who is like a family member Dr. Jacqueline Y. Miller, an American entomologist who has worked on Lepidoptera, especially Castniidae, for nearly 40 years. However, her most important legacy has been her guidance to lepidopterists throughout the world.

##### Included species.

*Millerana* is established as a new genus to include *Milleranaarbosioides* Dognin originally belonged to *Gaujonia*. Additionally, five new species are recognized that were formerly confused with *M.arbosioides* because of similarities in their wing markings: *Milleranaaustini* sp. nov., *Milleranacajas* sp. nov., *Milleranacundinamarquensis* sp. nov., *Milleranamatthewsae* sp. nov., and *Milleranatigrina* sp. nov. However, the external and internal morphology plus DNA barcoding revealed they are distinct. Females are unknown.

##### Diagnosis.

*Millerana* is the most genetically distant genus in the *Gaujonia* genus group and is more closely related to *Gaujoptera* (Fig. 1). Externally, *Millerana* is similar to *Cicadoforma* and *Cicadomorphus*, but smaller in size and a slightly different forewing pattern. The genitalia valva is simple as for *Cicadoforma* and *Cicadomorphus*, but much wider, with relatively long protuberances at the apex and outer margin. The vesica has a band of spines surrounding the middle area. DNA barcoding reveals that *Millerana* is distant from the other genera: *Gaujoptera* (~ 9% divergent), *Cicadoforma*, and *Cicadomorphus* (~ 18%).

##### Description.

Orbicular spot well developed; reniform spot with small lunate marking. Forewing pale yellow with black scales, which form the forewing pattern. Hindwing with yellow scales restricted to veins and margin, extending to fringe. Antenna dark brown or brownish orange, serrate, with a stripe of pale-yellow scales on basal % of antenna. Mouthparts reduced; eyes covered by black interfacetal setae. Male genitalia: moderately sclerotized; valva wide, without clasper; apex small with a pointed extension; uncus broad, beak-like; aedeagus short, vesica with narrow band of spines around middle part of vesica.

##### Immature stages.

***Egg.*** Circular and flattened with micro-square cells formed by the chorion. ***Larva*.** Like many pantheines, there are five to seven instars. Late instars resemble larvae of the genus *Panthea*. Secondary setae on the abdominal tergites spine-like. White lines between the spiracles similar to those of Panthea. Thorax densely covered by secondary setae, with long setae on the prothorax covering head. ***Pupa*.** Similar to those of other species in the *Gaujonia* genus group, but remarkably smaller (see *Cicadomorphusfalkasiska* immature stages) (Bollino and Onore 2001; O. Mahecha-Jiménez, pers. comm.).

##### Biology.

*Milleranatigrina* is the only species known and is considered a pest of pine trees in Ecuador (Bollino and Onore 2001).

**Figures 74–79.**
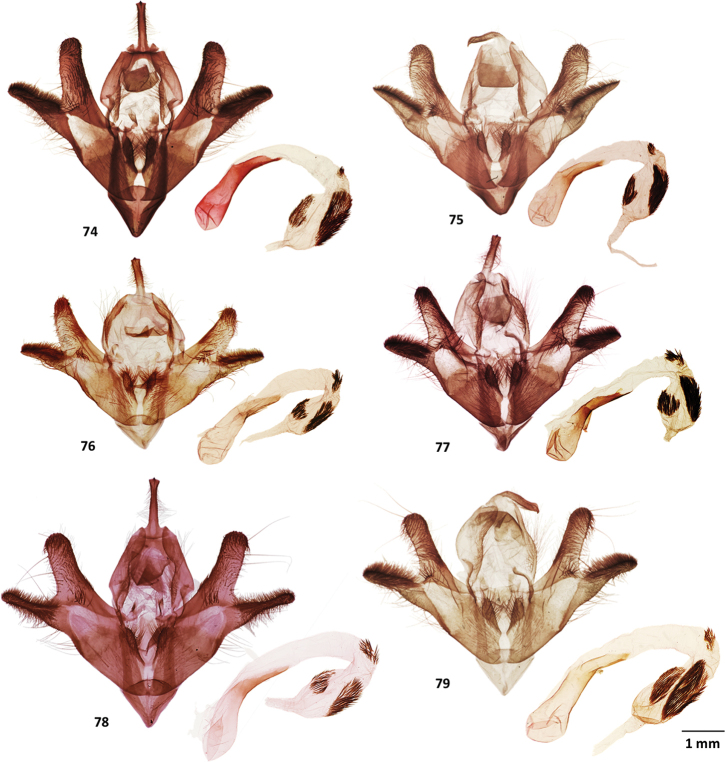
Male genitalia of *Oculicattus* species **74***Oculicattusinca*, holotype, MGCL, Sierra Siberia, Bolivia **75***O.boliviana*, holotype, MGCL, Sierra Siberia, Bolivia **76***O.uturunku*, holotype, MGCL, Morona, Ecuador **77***O.brehmi*, holotype, FSU, Loja, Ecuador **78***O.raizae*, holotype, MGCL, Tolima, Colombia **79***O.schmidti*, holotype, MGCL, Pasco, Peru.

### Key to species of the genus of *Millerana* based on adult male morphology

**Table:** 

1	Valva relatively square-shaped (Figs 70–73)	**2**
–	Valva petal-like (Fig. 69)	***M.cundinamarquensis***
2	Phallus with a band of spines on vesica close to base and another small patch of small spines apically (Figs 71–73)	**3**
–	Phallus with only a band of spines on vesica close to base (Fig. 70)	***M.tigrina***
3	Antenna black; forewing with a small orbicular spot (Figs 9, 37, 38, 42)	4
–	Antenna orange; forewing with a large orbicular spot (Fig. 39)	***M.matthewsae***
4	Cucullar region presenting outer margin with protuberances (Figs 9, 72)	**5**
–	Cucullar region without protuberances on the outer margin (Fig. 73)	***M.austini***
5	Forewing with a small and elongated orbicular spot; valva with square-shaped apex (Figs 9, 37)	***M.arbosioides***
–	Forewing with orbicular spot rounded; valva with rounded apex (Figs 42, 72)	***M.cajas***

#### Millerana arbosioides

Taxon classificationAnimaliaLepidopteraNoctuidae

(Dognin) comb. nov.

007255D1-ABAB-5C75-8C70-9E8F3DCC0A37

Figs 9 , 37 , 94

Gaujonia arbosioides Dognin, 1894: 87.

##### Type material.

***Holotype*** ♂, **Ecuador**: “*Gaujonia arbosioides* type ♂ Dgn. / ♂ 2700 mètres altitude, près Loja (Chonta-Cruz), 24 juin 1893 / *Gaujonia arbosioides* type ♂ Dogn., Hmpsn 15.10.12 / 1994 / Type No. 30908 U.S.N.M. / Genitalia slide m, Franclemont USNM 33565” coll. P. Dognin. Deposited in USNM. **Additional examined specimens** (2 ♂, MGCL): **Ecuador**: Ecuador, Carachi Prov., El Angel Ecological Reserve, 0°42'37"N, 78°00'12"W, 06.11.2012, H = 3560 m, Exped. Ron Brechlin & Victor Sinyaev.

##### Etymology.

Paul Dognin likely name this species *arbosioides* in reference to its similarities to *Gaujoniaarbosi*, which was described previously.

##### Diagnosis.

*Milleranaarbosioides* is closely related to three species: *M.austini*, *M.cajas*, and *M.cundinamarquensis*; however, *M.arbosioides* can be recognized by the black antenna and haustellum. Forewing length in males is 16–18 mm. *Milleranaarbosioides* shares more external characters with *M.cundinamarquensis*, but it can be distinguished from it by the antemedial line, which is well developed in *M.arbosioides*, the reniform spot is better defined, and the lunate marking is small; these features in *M.cundinamarquensis* are barely visible, but it presents four black tufts in the middle of 1A–4A, whereas *M.arbosioides* has three on A1–3A. The genitalia in *M.arbosioides* have a semi-squared cucullar area and some protuberances in the outer margin that are present in *M.tigrina* and *M.cajas* as well however, both species differ from *M.arbosioides* by their apexes that are rounded and their outer margins of the cucullar area, which are straighter.

##### Immature stages.

Unknown.

##### Distribution.

The species is known only from coniferous forests in Ecuador at very high elevations ca. 2500–3500 m (Fig. 94).

##### Biology.

Unknown.

##### Remarks.

One of the additional examined specimens was in very poor condition and only identifiable by genitalia examination.

#### Millerana austini sp. nov.

Taxon classificationAnimaliaLepidopteraNoctuidae

A3B3851B-3FEF-5986-AAC7-A74D44BE7CE4

http://zoobank.org/FA2F5CB1-1F31-4006-9DA5-BA7054D91D2D

Figs 38 , 73 , 94

##### Type material.

***Holotype*** ♂, **Ecuador**: Ecuador, Cotopaxi Prov., Minitrack Station paramo S of Machachi, 12,000’, 10 Apr. 1958, coll. R. W. Hodges. Deposited in CUIC. ***Paratypes*** (4 ♂, CUIC): **Ecuador**: Same collecting data as holotype.

##### Etymology.

The species was named after my friend Kyhl Austin, an American lepidopterist, who found this species in the dark corners of the CUIC.

##### Diagnosis.

*Milleranaaustini* is similar to *M.cajas*, but it can be differentiated by the antemedial line, which is formed by a single line, whereas *M.cajas* has two. The male genitalia are without protuberances on the outer margin, except for two extensions; one near the apex and the other on the lobe; they are similar to those of *M.tigrina*; however, the outer margin of the valve is concave in *M.tigrina*.

##### Description.

***Head*.** Palp black with a small line with pale yellow underneath; frons yellow. ***Thorax*.** Dark yellow with dots in black. ***Wings*.** Forewing dark yellow with black pattern; length 17–19 mm. All lines well developed with exception of terminal line; orbicular spot circular; reniform spot incomplete, with a small lunate marking; V-shaped mark on base of CuA2 thick, black; hind wing whitish orange, veins yellowish orange. ***Leg.*** Prothoracic legs black with some yellow spots on joints; mesothoracic legs with some black dots on tibia; metathoracic legs yellow. ***Abdomen*.** Paler yellow than thorax; small tufts in black from 1A–2A. ***Male genitalia*.** Cucullar area wide; apex and lobe with small extensions; outer margin flat; sacculus notably wide; tegumen broad; juxta inverted, triangle-shaped; aedeagus 3 ⅓ × longer than wide; vesica 1 ½ × wider than aedeagus, gradually reduced at tip; band with large spines and thick patch of spines near apex.

##### Immature stages.

Unknown.

##### Distribution.

All specimens were found in a cloud forest at the north-central region of Ecuador at high elevations above 3500 m (Fig. 94).

##### Biology.

Unknown.

##### Remarks.

Holotype (Fig. 38) and paratypes in perfect condition.

#### Millerana cajas sp. nov.

Taxon classificationAnimaliaLepidopteraNoctuidae

823F30C1-D06E-5F53-BF65-CF12EC4D9CE2

http://zoobank.org/0CC0DBCA-3428-474C-B339-50C24A2776FA

Figs 6 , 42 , 72 , 94

##### Type material.

***Holotype*** ♂, **Ecuador**: Ecuador, Azuay Prov., Cajas Nat.Park, Road Cuenca-Pto Inca, 2°46'50"S, 79°10'58"W, 28.11.2012, H = 3615m, Exped. Ron Brechlin & Victor Sinyaev / UF, FLMNH, MGCL 1049111. [DNA voucher MGCL-NOC-65294] deposited in MGCL. ***Paratype*** (1 ♂, MGCL): **Ecuador**: Same collecting data as holotype.

##### Etymology.

The only two specimens found in El Cajas National Park, Ecuador, are known.

##### Diagnosis.

*Milleranacajas* is similar to *M.austini*. The best way to identify *M.cajas* externally is to examine the medial and postmedial lines, which are fused on the forewing. Moreover, the genitalia are very distinctive. The valva is enlarged, especially in the costal region; *Milleranacajas* has a wide apical protuberance on the valve, similar to that of *M.matthewsae*; the vesica has a distal diverticulum surrounded by a spine band.

##### Description.

***Head*.** Palp short, black and yellow, except last segment that is black with some small yellow scales; frons yellow. ***Thorax*.** Covered with light lemon-yellow hair-like scales with some black spots. ***Wing*.** Light lemon yellow with black and gray pattern; forewing length: male 18–20 mm; forewing antemedial, medial, and subterminal lines well developed; whereas basal, postmedial, and terminal lines defined by small dots; medial line wide; orbicular spot slightly flattened; reniform spot large, incomplete, with lunate marking at base of M1 cell; V-shaped mark at base of CuA2 narrow; medial and postmedial lines fused by black scales between V-shaped mark in CuA2 and fold; hindwing whitish yellow with veins whitish orange. ***Leg.*** Prothoracic legs black with fuscous joints; mesothoracic and metathoracic legs yellow with a black spot on tibia; tarsi black. ***Abdomen*.** Paler yellow than remainder of body; tuft on middle area on A1–A5; A1–A3 black and A4–A5 yellow. ***Male genitalia*.** Cucullus wide, mainly in on costal area; apex with a wide extension followed by some protuberances on outer margin; saccular region wide; tegumen wide; juxta dentate, slightly curved on upper side; aedeagus 3 ½ × longer than wide; opening larger than rest of aedeagus; vesica long with a distal diverticulum, which is surrounded by a band of long spines; medial area with a large patch of minute spines.

##### Immature stages.

Unknown.

##### Distribution.

This species was found in a deciduous forest at a high elevation in southwestern Ecuador ca. 3600 m (Fig. 94).

##### Biology.

Unknown.

##### Remarks.

Holotype (Fig. 42) with the right hindwing and the left forewing slightly broken; the abdomen has a spot of dead fungi; the frons has pink marker stains accidentally applied at the place where it was previously deposited. The left wings are missing in the paratype.

#### Millerana cundinamarquensis sp. nov.

Taxon classificationAnimaliaLepidopteraNoctuidae

93F5844A-E5B5-51A7-A437-2746F1C51789

http://zoobank.org/E138732D-E065-4421-8BDD-C51F6494882F

Figs 39 , 69 , 94

##### Type material.

***Holotype*** ♂, **Colombia**: Colombia, Cundinamarca, Municipio Guasca, Alto El Paramo, 04°53'44"N, 73°45'46"W, 29–31.12.2017, 3250 m., Leg Viktor Sinjaev and Juan Machado / UF, FLMNH, MGCL 1049040. [DNA voucher MGCL-NOC-65224] deposited in MGCL. **Additional examined specimens** (1 ♂, MGCL): **Colombia**: same collecting data as holotype.

##### Etymology.

The name is derived from the place (Department of Cundinamarca in Colombia) where this species was found.

##### Diagnosis.

Compared with other species in *Millerana*, *M.cundinamarquensis* differs in the genitalia, in which the valva is petal-like, whereas the other two species have a rectangular valva.

##### Description.

***Head*.** Palpi short, divided by black hair-like scales on upper side and yellow underneath; last segment coated with a mix of black, white, and yellow scales; frons with sulfur-yellow scales. ***Thorax*.** Covered by sulfur-yellow scales with some large black spots. ***Wing*.** Forewing length: male 16–18 mm; forewing sulfur-yellow with black scales defining transverse lines; antemedial, medial, and subterminal lines barely developed; basal, postmedial, and terminal lines defined by series of dots on veins; orbicular spot well-defined, oval; reniform spot blurry, with a small triangular lunate marking; CuA2 with a black V-shaped mark at base, with inferior line longer than superior line; hindwing with yellowish orange veins; fringe with pale-yellow hair-like scales; base of M2+M3 rounded. ***Legs*.** Yellow except prothoracic legs, which are black with pale yellow spots. ***Abdomen*.** Pale yellow with light-gray hair-like scales; four black tufts in the middle of A1-A4; a small pale-yellow tuft on A8 at terminus. ***Male genitalia*.** Cucullar area with rounded apex, apex extension claw-like; costa ovoid, covered with setae; saccular area wide; tegumen narrow and rounded; juxta with concave V-shaped depression on top; aedeagus 4 × longer than wide; opening to vesica % × total length of aedeagus; vesica long, 2 × wider than aedeagus, decreasing in width as it approaches to apex; a band of medium-size spines near basal area of vesica.

##### Immature stages.

Unknown.

##### Distribution.

Only two specimens are known from a cloud forest in Cundinamarca, Colombia (Fig. 94).

##### Biology.

Unknown.

##### Remarks.

Holotype well preserved (Fig. 39), but the other specimen, which was collected from the same location, was difficult to identify because it was practically destroyed. Thus, the identification was made by genital dissection.

**Figures 80–84.**
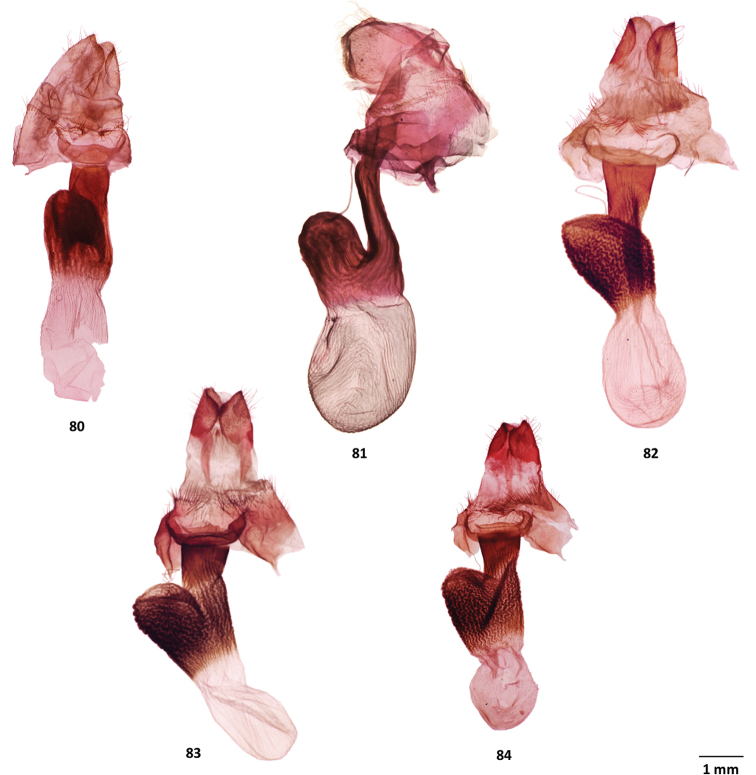
Female genitalia of *Cicadoforma* and *Cicadomorphus* species **80***Cicadoformaocelotus*, paratype, MGCL, Santander, Colombia **81***C.vau-nigrum*, CNC, El Junquito, Venezuela **82***Cicadomorphuslilianae*, paratype, FSU, Zamora-Chinchipe, Ecuador **83***C.falkasiska*, paratype, MGCL, Oxapampa, Peru **84***C.chuya*, paratype, MGCL, Cochabamba, Bolivia.

#### Millerana matthewsae sp. nov.

Taxon classificationAnimaliaLepidopteraNoctuidae

AE610BCE-43F8-50A2-9813-9339A5848954

http://zoobank.org/CCB1BFCF-B1ED-4ED9-A7B7-E86569702EC0

Figs 40 , 71 , 94

##### Type material.

***Holotype*** ♂, **Peru**: Peru, Ancash 7 km W Yanama, 09°02.0'S, 7°32.9'W, 03.03.2011, H = 3892 m, leg. Viktor Sinyaev & Alexander Poleschuk / UF, FLMNH, MGCL 1049106. [DNA voucher MGCL-NOC-65288] deposited in MGCL. ***Paratype*** (1 ♂, MGCL): **Peru**: Same collecting data as holotype. **Additional examined specimens** (3 ♂, MGCL): **Peru**: Same collecting data as holotype.

##### Etymology.

In honor of my colleague and friend Dr. Deborah Matthews-Lott, who has supported me during my travels in entomology, especially on Lepidoptera trips.

##### Diagnosis.

*Milleranamatthewsae* is closely related to *M.tigrina*; it has brownish orange antenna and can be differentiated by the wide well-defined pattern of foreign lines, and the large orbicular and reniform spots. The most distinctive character of the male genitalia is the costal margin of the valva is strongly curved upward.

##### Description.

***Head*.** Palp with long black and yellow hair-like scales; frons dark yellow. ***Thorax*.** Dark yellow with some black patches on dorsal area; ***Wing*.** Forewing length: male 18–20 mm; forewing paler yellow coloration than thorax; antemedial, medial, and subterminal lines complete; postmedial line almost complete except in area between cells of CuA1 and half of CuA2; V-shaped mark at base of CuA2 with upper line longer than lower line; terminal area covered by scattered black scales except in M3 and CuA1 cells; orbicular spot very large, barely touching top and bottom of discal cell; reniform spot with large line marking inside of discal cell; hindwing white with veins and fringe pale yellow. ***Leg.*** Prothoracic and mesothoracic legs brownish orange and black with joints yellow; metathoracic legs yellow. ***Abdomen*.** Yellow, paler than remainder of body; dorsally with thin black tufts in middle of A1–A6 and with dark yellow tufts at A2–A3. ***Male genitalia*.** Cucullus wide and curved upward on costal margin; apex rounded; some protuberances in the outer margin with the upper one the largest and curved downward; sacculus wide and short; upper side of juxta has a shallow concave depression; tegumen narrow laterally; aedeagus narrow, ⅓ × longer than vesica; vesica long rounded basally; Transverse band of spines near middle of vesica.

##### Immature stages.

Unknown.

##### Distribution.

This species has been collected only in the western zones of Peru at very high elevations almost 4000 m (Fig. 94).

##### Biology.

Unknown.

##### Remarks.

The holotype (Fig. 40) and the paratype have both hindwings slightly broken. The other specimens were almost destroyed with only the thorax and forewings remaining.

#### Millerana tigrina sp. nov.

Taxon classificationAnimaliaLepidopteraNoctuidae

F0B099DA-E73D-5AB1-B021-85541549686F

http://zoobank.org/822C7DB2-1AFB-4CA2-9B01-0129C42AC289

Figs 41 , 70 , 94

Gaujonia arbosioides Hampson, 1913 (nec. Dognin): pl. 235, fig. e2.
Gaujonia arbosi Gara & Onore, 1989 (nec. Dognin): 128, figs 58, 59.

##### Type material.

***Holotype*** ♂, **Ecuador**: Ecuador, Carchi road Tulcan – El Chical, 0°48'19"N, 78°00'10"W, 12–13. II 2013, 3400 m, leg. Sinjaev & Romanov / UF, FLMNH, MGCL 1049105. [DNA voucher MGCL-NOC-65287] deposited in MGCL. ***Paratype*** (1 ♂, MGCL): **Ecuador**: Ecuador, Azuay Prov., Cajas Nat. Park, Road Cuenca-Pto Inca, 2°46'50"S, 79°10'58"W, 28.11.2012, H = 3615 m, Exped. Ron Brechlin & Victor Sinyaev.

##### Etymology.

The name refers to the tigrina, *Leopardusguttulus* (Hensel), in keeping with the wild feline names, as with the other genera, since they are known as “the jaguar moths.”

##### Diagnosis.

*Milleranatigrina* can be distinguished from *M.matthewsae* by the large, well-rounded orbicular spot. *Milleranatigrina* also has distinctive genitalia, with the valva presenting a pronounced concavity on the outer margin, forming a moon-shaped crescent.

##### Description.

***Head*.** Palp covered with hair-like scales with dorsal side black and ventral side whitish yellow; last segment black with small whitish yellow dot internally; frons yellow. ***Thorax*.** Covered with yellow hair-like scales with black patches. ***Wing*.** Forewing length: male 17–19 mm; forewing yellow with black scales forming wing pattern; basal line barely visible; antemedial and medial lines disrupted at discal cell; reniform spot large, with small lunate marking in middle; orbicular spot rounded, almost as high as discal cell; two V-shaped marks, one at base of R1+R2, other one at base of CuA2; hindwing whitish silver with yellow fringe; veins yellow. ***Leg.*** Prothoracic and mesothoracic legs dark brown with some patches of black and pale yellow; metathoracic legs pale yellow with dark brown from tibia to claws. ***Abdomen*.** Covered with pale-yellow, hair-like scales; three small black tufts on A1–A3; a pale-yellow terminal tuft. ***Male genitalia*.** Outer margin of cucullar area concave, with two small protuberances, one subapical and lateral to apex and other one smaller, on lower end of lobe; saccular area wide; tegumen wider dorsally and squared; juxta concave posteriorly; aedeagus 3 × longer than wide; a diagonal opening to vesica ⅓ length of aedeagus; vesica almost as long as aedeagus; transverse band of spines near base.

##### Immature stages.

***Egg.*** Bright green; turns dark green close to emergence. ***Larva*.** Five to seven instars; body brown with black and pale-brown secondary setae arising from pink verrucae; diagonal pinkish white lines laterally between spiracles; spiracles white; thorax covered with secondary setae; two tufts of on each side of second tergite. ***Pre-pupa*.** Similar to last instar, but darker and with secondary setae shorter. ***Pupa*.** Dark brown.

##### Distribution.

The two specimens of *Milleranatigrina* were found in the highest elevations of the Andes in Ecuador (Fig. 94).

##### Biology.

Only one species is known in this genus, *Milleranatigrina*, which was discussed first by Bollino and Onore (2001). Adults are active throughout the year, but mainly during summer. Larvae resemble some species of the genus *Panthea*, which also feed on pine trees. Larvae have been observed to feed on *Podocarpusmagnifolius* J. Buchholz & N.E. Grayand and *Quercushumboldtii* Bonpl as well (O. Mahecha-Jiménez pers. comm.).

##### Remarks.

The type specimen (Fig. 41) has a small patch of dead fungi on the ventral side of the abdomen. The specimen from Azuay is poor condition but is still identifiable. This species was misidentified as the female of *Milleranaarbosioides* by Hampson (1913) (as *Gaujoniaarbosioides*) and Seitz (1919–1944). In addition, the larvae are reported as pine tree pests in Ecuador (Gara and Onore 1989), but was misidentified as *Gaujoniaarbosi*. Unfortunately, the pictures of the immature stages provided by O. Mahecha-Jiménez were not of good enough quality to be included in this revision.

#### Oculicattus gen. nov.

Taxon classificationAnimaliaLepidopteraNoctuidae

D11289FF-F773-5B33-BEA8-D3FDFFAAFB10

http://zoobank.org/5372CF2B-65A3-49D9-89DC-3270DA5E8035

##### Gender.

Masculine.

##### Type species.

*Gaujoniarenifera* Hampson, 1913. Catalogue of Lepidoptera Phalaenae in the British Museum 13: 385, 387, pl. 235, fig. 4.

##### Etymology.

*Oculicattus* refers to the reniform spot, which is black and surrounded with yellow scales, giving it the appearance of a cat’s eye.

##### Included species.

*Oculicattus* is a new genus established for *Gaujoniarenifera* (Hampson), which was misplaced in the genus *Gaujonia*. This genus also includes six new species, *Oculicattusboliviana* sp. nov., *Oculicattusbrehmi* sp. nov., *Oculicattusinca* sp. nov., *Oculicattusraizae* sp. nov., *Oculicattusschmidti* sp. nov., and *Oculicattusuturunku* sp. nov., which are morphologically and genetically distinct from the genus *Gaujonia*.

##### Diagnosis.

*Oculicattus* can be differentiated from *Gaujonia* externally by the presence of the large reniform spot in *Oculicattus*, as well as by other features (see *Gaujonia* diagnosis).

##### Description.

Sexually dimorphic in size, females larger than males; forewing and hindwing hyaline with sulfur-yellow and black scales covering veins and wing margins. Forewing with a small black or sulfur-yellow orbicular spot, sometimes barely perceptible; reniform spot elongated, outlined in black; elongated black central line surrounded by sulfur-yellow outline, except for *O.raizae* and *O.uturunku*, in which the spot is entirely black. Antenna filiform, brownish orange with a sulfur-yellow band on basal to three quarters of antenna; eyes with coppery interfacetal setae. Male genitalia slightly sclerotized; valva with saccular and cucullar regions separated, without clasper; uncus long and narrow ending in beak-like tip; vesica has spine patch; vesica wider than its base, which has two patches of spines with one patch of spines larger than other. Female genitalia medium sized; lightly sclerotized rectangular-shaped sterigma; appendix bursae elongate and rugose; corpus bursae for most species approximately half size of appendix bursae.

##### Immature stages.

Unknown.

##### Biology.

Unknown.

### Key to species of the genus of *Oculicattus* based on adult male morphology

**Table:** 

1	Forewing with reniform spot poorly defined and not outlined (Figs 49–51)	**2**
–	Forewing with well-developed and outlined reniform spot (Figs 43–48, 52, 53)	**3**
2	Thorax and forewing brownish yellow with brown pattern; valva with cucullar region wide and rounded apically; short aedeagus; wide patch of spines close to basal area (Figs 50, 51, 78)	***O.raizae***
–	Thorax and forewing black with some spots in sulfur-yellow; valva with cucullar area wide at basally and remarkably narrow apically; aedeagus elongate; thin patch of spines in basal area (Figs 49, 76)	***O.uturunku***
3	Saccular process tapered to a pointed apex (Figs 75, 79)	**4**
–	Saccular process is blunt or rounded (Figs 74, 77)	**5**
4	Forewing with a small black orbicular spot and a large reniform spot; aedeagus, short, wide (Figs 48, 75)	***O.boliviana***
–	Forewing with a small yellow orbicular spot and a narrow reniform spot; aedeagus long, narrow (Figs 45, 46, 79)	***O.schmidti***
5	Cucullus with same width overall; vesica long with both apical patches ovoid (Figs 10, 74)	**6**
–	Cucullar region wider at base than apex; vesica short with a truncated patch apically (Fig. 77)	***O.brehmi***
6	Ground color grayish yellow; thorax with gray patches dorsally; A3 on abdomen with large yellow dorsal tuft; vesica short with a small patch of spines near more basal that apical; (Figs 47, 74)	***O.inca***
–	Ground color pale brownish yellow; brownish black spots scattered on thorax dorsally; line of black and yellow tufts on A1–A3 on dorsum of abdomen; long vesica, with a large patch of spines near the middle point of the vesica (Figs 10, 43, 44)	***O.renifera***

#### Oculicattus boliviana sp. nov.

Taxon classificationAnimaliaLepidopteraNoctuidae

2BD9F0AD-3DB5-5112-A15F-FF03305598F9

http://zoobank.org/AC56D195-6D14-4D26-9090-3E3B3D8235EF

Figs 48 , 75 , 95

Gaujonia arbosi Gowin, 2017 (nec. Dognin): pl. 47 fig. 6.

##### Type material.

***Holotype*** ♂, **Bolivia**: Bolivia, Sierra Siberia, 16 km SE Pojo, 17°49.1'S, 64°42.5'W, 14.12.2009, H = 2308 m, leg/coll. Viktor & Svetlana Sinyaev + Alexei Zamesov. Deposited in MGCL. ***Paratypes*** (2 ♂, MGCL): **Bolivia**: Same collecting data as holotype (1 ♂);, La Higuera, 18°47.7'N, 64°12.1'W, 19–20.12.2009, H = 2050 m, leg/coll. Viktor & Svetlana Sinyaev + Alexei Zamesov (1 ♂).

##### Etymology.

This species is only found in Bolivia, hence the proposed name.

##### Diagnosis.

This species can be distinguished from *O.renifera*, and *O.schmidti* by the large lunate mark in the reniform spot, being lightning bolt shaped, and by the small orbicular spot. The male genitalia have the saccular process with a sharply pointed tip; uncus thin. *Oculicattusuturunku* and *O.boliviana* share almost identical mtDNA, however, distribution and biology, in addition to morphology demonstrate that they are different species.

##### Description.

***Head*.** Segments of palp divided in black upper side and yellow underside; two large black spots posterior to antenna. ***Thorax*.** Almost entirely light yellow with some small black spots on dorsal area. ***Wing*.** Light yellow with wide black lines; forewing length: male 17–19 mm; forewing yellow, like rest of body, with black stripes defining pattern; reniform spot with large lightning bolt-like lunate marking; orbicular spot black, small, outlined in pale yellow; open V-shaped mark on CuA2 at base wide; fringe on hindwing light yellow with black terminal line, interrupted at the veins; posterior margin with fringe whitish yellow; vein yellow with three lines on each vein from M1 to CuA2. ***Leg.*** Prothoracic leg black with tibia and metatarsi yellow, whereas tarsi black, even at joints. ***Abdomen*.** Bright yellow with dorsal region clothed with dark brown scales; dorsal abdomen with yellow tufts and a thin black line on A1–A5. ***Male genitalia*.** Cucullar area wide, densely covered by hair-like setae; apex tapered; saccular area relatively narrow, ends in sharp process with a sharply pointed tip; saccular process with setae mainly confined to upper side; saccus V-shaped, considerably flat on tip; juxta flat on upper side and narrow on under side; tegumen wide; uncus thin; aedeagus 1 ⅔ × longer than wide; vesica base ½ × as long as vesica; two oval-shaped subapical spines patches, with small cluster of spines is near the middle of the dorsal wall of the vesica.

##### Immature stages.

Unknown.

##### Distribution.

The specimens were collected in south-central Bolivia at high elevations above 2000 m (Fig. 95).

##### Biology.

Unknown.

##### Remarks.

Holotype (Fig. 48) and paratypes are in perfect condition. This species was originally misidentified as *Gaujoniaarbosi* by Gowin (2017), which is endemic to Ecuador, and is here assigned to a different genus and it was photographed at Laguna Verde-Comarapa and Achira Arriba in Bolivia.

**Figures 85–90.**
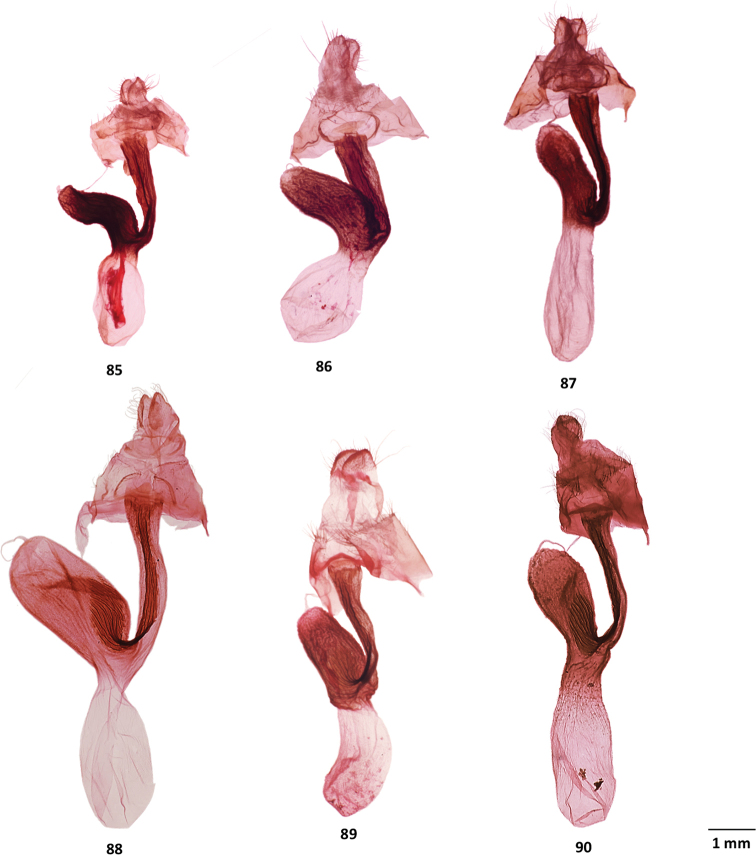
Female genitalia of *Gaujonia* and *Oculicattus* species **85***Gaujoniaarbosi*, MGCL, Napo, Ecuador **86***G.chiqyaq*, holotype, MGCL, Marinio, Colombia **87***G.kanakusika*, paratype, MGCL, Cundinamarca, Colombia **88***Oculicattusrenifera*, MGCL, Cusco, Peru **89***O.brehmi*, paratype, MGCL, Napo, Ecuador **90***O.raizae*, paratype, MGCL, La Paz, Bolivia.

#### Oculicattus brehmi sp. nov.

Taxon classificationAnimaliaLepidopteraNoctuidae

414F14EE-7ADC-5DA1-9F94-2DBB83F98FC6

http://zoobank.org/8F0D6FD9-CCCF-4A0A-8EE0-B17AB28E16A4

Figs 52–55 , 77 , 89 , 95

##### Type material.

***Holotype*** ♂, **Ecuador**: Ecuador, 8 km SE of Loja, Parque Nacional Podocarpus Cajanuma, mont. Rainforest, Blacklight 2 × 15W (50), 04°06.86'S, 79°10.48'W, 20.ix.2008, 2897 m, Florian Bodner leg / DNA Barcode run 2010, COI-5P marker, University of Guelph / Arcec 32176. [DNA voucher Arcec 32455] deposited in FSU. ***Paratypes*** (1 ♂, FSU) : **Ecuador**: Ecuador, Zamora-Chinchipe Parque Nacional Podocarpus, Cerro Toledo,Elfin Forest, Blacklight 2 × 15W (70), 04°23.13'S, 79°07.11'W, 6.II. 2013, 19.00–22.00 h, 2938 m, Gunnar Brehm leg. / DNA Barcode run 2013, COI-5P marker, University of Guelph / leg sampled in ethanol G. Brehm, Green vial caps / [Arcec 30239] (1 ♂). (4 ♂, 2 ♀, MGCL): **Ecuador**: Ecuador, Napo + 10 km Papallacta, 2730 m, 13–15 Sep. 1982, coll. N. Venedictoff (1 ♂, 2 ♀); Ecuador, Napo, Cosanga, 2150 m, 1 Feb. 1976, coll. N. Venedictoff (3 ♂). **Additional examined specimens** (2 ♂, 1 ♀ NJD): **Ecuador**: Ecuador, Eastern slopes of the Andes, east of Quito near Cosanga, Yanayacu Biological Research Station, 2100 m, 27 Aug. 2013, “YAN13_0136”, N. J. Dowdy (1 ♂); Ecuador, Eastern slopes of the Andes, east of Quito near Cosanga, Yanayacu Biological Research Station, 2100 m, 27 Aug. 2013, “YAN13_0056”, N. J. Dowdy (1 ♂) ; Ecuador, Eastern slopes of the Andes, east of Quito near Cosanga, Yanayacu Biological Research Station, 2100 m, 27 Aug. 2013, “YAN13_0072”, N. J. Dowdy (1 ♀).

##### Etymology.

The name is in honor of a great lepidopterist, Dr. Gunnar Brehm, who loaned some specimens for this research.

##### Diagnosis.

*Oculicattusbrehmi* is the smallest species in this genus; pattern formed by relative pale, thin markings. Orbicular spot small, black. There V-shaped mark at the base of CuA2 is small. Male genitalia have a tapered cucullar region, wider at the base; the saccus smaller and more tapered than in other species, and the saccular process is the second shortest in the genus with only *O.uturunku* being shorter than *O.brehmi*.

##### Description.

***Head*.** Wide, palp large, with last segment marbled in light yellow and black; frons pale yellowish gray; female similar to male, even in coloration. ***Thorax*.** Covered with dark sulfur-yellow scales and with small gray spots on dorsum. ***Wing*.** Pale yellow with pattern of thin dark gray lines; forewing length: male 16–18 mm; female: 21–23 mm; forewing with thin dots and stripes forming lines; lunate marking in reniform spot narrowly outlined by a thin yellow line; orbicular spot black, small; V-shaped mark at base of CuA2 small; hindwing with fringe yellow with long yellow lines between veins terminally. ***Leg.*** Prothoracic leg brown with joints pale yellow; mesothoracic legs marbled in brown and yellow, tarsi brown with each joint yellow; metathoracic legs yellow. ***Abdomen*.** Pale yellow with dorsal area gray; dorsal tufts sulfur-yellow along middle of abdomen, smaller on A1–A2, whereas A5–A8 are wide and combined with gray scales. ***Male genitalia*.** Cucullus tapered with small rounded apex and base wide, heavily covered with setae; sacculus wide with process long and densely clothed with setae; saccus V-shaped, and more narrowly tapered towards the end; juxta wide deeply concave posteriorly, , with the outer edges sharply pointed; tegumen wide; aedeagus 1 ¼ × length of vesica to medial cluster of spines; vesica with small patch of spines near middle; apical part of vesica bulbous, with large tapered patch of spines on each side. ***Female genitalia***. anal papilla rounded posteriorly; posterior apophysis ⅓ × longer than anal papilla; anterior apophysis ⅔ × shorter than posterior apophysis; sterigma crescent moon shaped; corpus bursae ¼ × longer than appendix bursae.

##### Immature stages.

Unknown.

##### Distribution.

This species has been recorded only in Ecuador (Fig. 95).

##### Biology.

Unknown.

##### Remarks.

Holotype (Fig. 52) and paratypes are in good condition; specimens from NJD were soaked in 100% ethanol with only the wings in good condition; they were kept in – 80 °C for molecular DNA analyses, so they are not included with the type series.

#### Oculicattus inca sp. nov.

Taxon classificationAnimaliaLepidopteraNoctuidae

B941DDAE-F551-544F-8905-8993B8BFF808

http://zoobank.org/7DFAA7B1-E42A-4864-AB61-8E071D5A5C81

Figs 47 , 74 , 95

##### Type material.

***Holotype*** ♂, **Peru**: Peru, Department Cuzco, Manu Park, San Pedro, 1800 m, Mar. 1997, coll. local people / UF, FLMNH, MGCL 1049174. [DNA voucher MGCL-NOC- 65359] deposited in MGCL. ***Paratypes*** (8 ♂, MGCL): **Peru**: Same collecting data as holotype (4 ♂); **Bolivia**: BoliviaA, Sierra Siberia 18 km SE Pojo, 17°50.2'S, 64°42.1'W, 12–13.12.2009, H = 2442 m, leg/coll. Viktor & Svetlana Sinyaev + Alexei Zamesov (2 ♂); Bolivia, La Paz, Cotapata, 16°16.5'S, 67°51.6'W, 24.10.2010, H = 3200 m, leg. Viktor Sinyaev & Oleg Romanov (1 ♂); Bolivia, La Paz, Santa Rosa de Lima, 16°23.6'S, 67°41.8'W, 20–22.10.2010, H = 1550 m, leg. Viktor Sinyaev & Oleg Romanov (1 ♂).

##### Etymology.

This is species is named after the Inca Empire, which originated in the area around Cusco.

##### Diagnosis.

*Oculicattusinca* is similar to *O.renifera*; however, there are some morphological features that are useful to differentiate them. *O.inca* is slightly smaller, darker in coloration, the orbicular spot smaller, and wing lines are thinner than in *O.renifera*; the lunate marking in the reniform spot is not outlined in yellow. Genitalia have a wider cucullus; the juxta is flattened posteriorly, whereas in *O.renifera* the posterior margin has a V-shaped invagination. The DNA barcodes differ by 5% between the two species.

##### Description.

***Head*.** Last segment of palp divided in black upper side and yellow under side; frons yellow with some gray scales. ***Thorax*.** Grayish yellow with some small gray patches. ***Wing*.** Forewing length: male: 19–21 mm; forewing grayish yellow with markings wide; orbicular spot small, elongated, grayish yellow, outlined with black scales and with some black scales inside orbicular spot; reniform spot with lunate marking not outlined; V-shaped mark short; hindwing with fringe black, vein ends yellow, with some black scales on vein CuA2. ***Leg.*** Prothoracic and mesothoracic legs black anteriorly, yellow posteriorly, whereas metathoracic legs all yellow. ***Abdomen*.** Dorsal area brown with some yellow scales between each tergite; a large yellow tuft on A3; last abdominal segment with long yellow scales. ***Male genitalia*.** Cucullar and saccular regions large; saccus triangular; juxta V-shaped with posterior part flattened; vesica 1 ½ × size of aedeagus; Vesica with small patch of spines near middle of vesica; bulbous apical part of vesica with large dense patch of spines posteriorly and smaller, less dense patch anteriorly.

##### Immature stages.

Unknown.

##### Distribution.

Broadly distributed in the cloud forests in Peru and Bolivia (Fig. 95).

##### Biology.

Unknown.

##### Remarks.

Holotype is in perfect condition (Fig. 47). This species was originally labeled as *Oculicattusrenifera*, but DNA barcoding showed that the two species are genetically distinct.

#### Oculicattus raizae sp. nov.

Taxon classificationAnimaliaLepidopteraNoctuidae

2E45AF37-9CF4-5CA0-BABB-FA0B0513F8B6

http://zoobank.org/BD31472B-72CC-4B58-B011-ED8E5339F5D4

Figs 50 , 51 , 58 , 78 , 90 , 95

##### Type material.

***Holotype*** ♂, **Colombia**: Colombia, Tolima, Nevado del Tolima, 4°36'02’’N, 75°19'51’’W, 2600 m, 05–07.12.2013, legit Victor Sinyaev & Mildred Márquez Martinez / UF, FLMNH, MGCL 1049101. [DNA voucher MGCL-NOC- 65284] deposited in MGCL. ***Paratypes*** (6 ♂, 1 ♀, MGCL): **Ecuador**: Same collecting data as holotype (2 ♂); Ecuador, Carchi, El Angel Ecological Reserve, 0°45'31"N, 78°01'40"W, 7–8. XI 2012, 3320 m, leg. Sinjaev & Romanov (1 ♂); Ecuador, Carchi prov., El Moran, 0°45'50"N, 78°02'38"W, 1–3.05.2012, H = 2940 m, Exped. Ron Brechlin & Victor Sinyaev (1 ♂); Ecuador, Pichincha, Camping Bella Vista, 2230 m, 0°00'41"S, 78°41'17"W, 19.XII.2012–7.I.2013, leg. Sinjaev & Romanov & [coll.] Dr. R. Brechlin (1 ♂); Ecuador, Pichincha, Quito/Chiriboga, K40, 2480 m, 22 Mar. 1982, coll. N. Venedictoff (1 ♀); **Peru**: Peru-Junin Near Calabaza vill., 11°30.4'S, 74°51.7'W, 20.12.2010, H = 2722 m, leg/coll. Viktor & Svetlana Sinyaev + Vladimir Izerskiy (1 ♂); **Bolivia**: BOLIVIA, La Paz, Death road Coroico, 16°18.3'S, 67°48.8'W, 28–30.10.2010, H = 3060 m, leg. Viktor Sinyaev & Oleg Romanov (1 ♀). **Additional examined specimens** (1 ♂, ZSM): **Ecuador**: Ecuador, Anden Oskordillere, Prov. Pichincha, Tandayapa, Km. 3 S (Bellavista, Lodge), 2310 m, 00°03'694"N, 78°40'929"W 1–20.ix.2012, Dietl Monika + Stefan &, R. Beck leg. / BC ZSM Lep 65183 / coll. G. Behounek, grafing bei Müchen / “*Gaujonia renifera* ♂” (1 ♂).

##### Etymology.

The species is dedicated to my wife, Raiza Castillo, for her love and support since the beginning of my career.

##### Diagnosis.

*Oculicattusraizae* is closely related to *O.uturunku*; they differ from the other *Oculicattus* species by the darker color, and the unusual reniform spot that is completely brown or black. *Oculicattusraizae* can be identified by its brownish yellow coloration, and the brown forewing pattern. In the male genitalia the cucullus is longer and wider than in *O.uturunku*. Additionally, DNA barcodes are ca. 4% different.

##### Description.

***Head*.** Palp reduced in size; dorsal surface covered by brown scales, ventral surface by dark sulfur-yellow scales; frons covered by dark sulfur-yellow scales, mixed with black; female similar to male externally. ***Thorax*.** Dark sulfur-yellow with some brown patches. ***Wing*.** Forewing with dark sulfur-yellow and brown scales covering venation and margins; forewing length: male: 22–24 mm; female: 27–29 mm; antemedial, postmedial, and subterminal lines not defined; orbicular spot small, poorly-defined oblong brown spot; reniform spot brown, narrow, and barely defined; base of CuA2 with Y-shaped mark large, brown; a brown line across fold on cell at CuA2; hindwing hyaline with some dark sulfur-yellow scales on venation, but more notable along margins; a Y-shape mark at base of CuA2; some small brown lines scattered through M3+CuA1+CuA2. ***Leg*.** Dark sulfur-yellow with brown patches on anterior area of prothoracic legs, which is lighter on mesothoracic and metathoracic legs. ***Abdomen*.** Clothed in grayish yellow hair-like scales with white scales at terminus and with a dark sulfur-yellow tuft on A8. ***Male genitalia*.** Cucullus wide, parallel sided with the apex rounded; costal margin swollen basally; sacculus triangular with saccular process entirely coated with setae; tegumen wide; saccus broadly V-shaped; juxta small with parallel sides and a V-shaped notch posteriorly; aedeagus 3 × longer than wide, with a wide opening to vesica ca. ¼ × total length of aedeagus; vesica with basal area ca. same width that of aedeagus, and it is ventrally curved; basal area with a large patch of spines; vesica with two patches of spines on each side, one small slightly beyond middle of dorsal wall of the vesica, and truncated anteriorly, whereas other larger apically narrow, and broader towards the aedeagus. ***Female genitalia***. Small and truncated apically; anal papilla with posterior apophysis ¼ × shorter than anal papilla; anterior apophysis short; rectangular sterigma that is fused above ostium; ductus bursae wide and long; appendix bursae ⅔ × shorter than corpus bursae which is partially sclerotized at base.

##### Immature stages.

Unknown.

##### Distribution.

Recorded from cloud forests in Colombia, Ecuador, Peru, and Bolivia in middle and high elevations from 2000 m to above 3000 m (Fig. 95).

##### Biology.

Unknown.

##### Remarks.

A specimen of *Oculicattusraizae* was misidentified as *G.renifera* by G. Behounek and posted on Barcode Of Life Data System v4 (www.barcodinglife.org). Holotype (Fig. 50) and paratypes are well preserved.

#### Oculicattus renifera

Taxon classificationAnimaliaLepidopteraNoctuidae

(Hampson) comb. nov.

11DA460F-FD6A-592C-84F2-14E792767D9B

Figs 7 , 10 , 43 , 44 , 56 , 57 , 88 , 95

Gaujonia renifera Hampson, 1913: 387, pl. 235, fig. 4.

##### Type material.

***Holotype*** ♂, **Peru**: “*Gaujonia renifera* type ♂ Hmpsn. / Agualani S. E. Peru, 9000 ft, 05’ May, G. Ockenden / 1908-159 / Noctuidae ♂ genitalia slide No. 5208 / NHMUK 010917655”, coll. G. Hampson. Deposited in NHMUK. **Additional examined specimens** (8 ♂, MGCL): **Peru**: PERU-JUNIN Near CALABAZA vil., 11°30.5'S, 74°49.4'W, 1–2.02.2011, H = 2137 m, leg. Viktor Sinyaev & Alexander Poleschuk (1 ♂); Peru, Department Cuzco, Manu Park, San Pedro, 1800 m, Mar. 1997, coll. local people (2 ♀); Peru, Cusco, Wayqecha Biological Station, 2950 m, 29 Oct 2010, coll. C. V. Covell Jr / FLMNH, MGCL 1049183 / DNA voucher MGCL-NOC- 65325 (1 ♂); **Bolivia**: BOLIVIA, SIERRA SIBERIA, 16 km SE Pojo, 17°49.1'S, 64°42.5'W, 16–17.01.2010, H = 2308 m, leg/coll. Viktor & Svetlana Sinyaev + Alexei Zamesov (2 ♂); BOLIVIA, La Paz, Cotapata, 16°16.5'S, 67°51.6'W, 24.10.2010, H = 3200 m, leg. Viktor Sinyaev & Oleg Romanov (1 ♂); BOLIVIA, La Paz, Santa Rosa de Lima, 16°23.6'S, 67°41.8'W, 20–22.10.2010, H = 1550 m, leg. Viktor Sinyaev & Oleg Romanov (1 ♂).

##### Etymology.

George F. Hampson probably named this species *renifera* based on the characteristic reniform spot.

##### Diagnosis.

Regarding *Ocullicatusrenifera*, there is one species that is particularly similar to it (*O.inca*); however, *O.renifera* can be identifiable by its remarkably large yellow orbicular spot. The male is pale yellow, brighter yellow in the female. The forewing length in males is 19–21 mm and females 24–26 mm. Palp reduced, black with white tips; frons yellow and gray; antenna dark orange; male thorax dark yellow with scattered brownish black spots. Pattern on the forewing in male is created by black small lines and dots; the reniform spot has slightly curved and wide lunate marking. The hindwing has dark scales on the veins. The abdomen is brown with yellow scales dorsally, differing from *O.inca* in having a line of black tufts over the middle of the dorsum of the abdomen; black tufts on A1–A3 combined with yellow scales. Genitalia of male with the cucullar region narrow and the apex rounded; saccular area with the process almost same size that the cucullus; aedeagus long, almost the same size of the vesica; large patch of spines on the base of the vesica; the two spine patches large and wide similar size covering ¾ of the vesica. Genitalia in female with sterigma relatively open, with the corpus bursae almost the same size as the appendix bursae.

##### Immature stages.

Unknown.

##### Distribution.

*Oculicattusrenifera* is restricted to Peru and Bolivia (Fig. 95).

##### Biology.

Unknown.

##### Remarks.

Many of the new species are similar to this species making the process of identification difficult. The holotype is in perfect condition (Fig. 10).

#### Oculicattus schmidti sp. nov.

Taxon classificationAnimaliaLepidopteraNoctuidae

B4251AC3-23D7-53C9-8CD0-D0E8E39C9BCD

http://zoobank.org/3A0958E1-36B7-4885-AB26-E4C210B2BB1D

Figs 45 , 46 , 79 , 95

##### Type material.

***Holotype*** ♂, **Peru**: Peru-Pasco 15 km SW Oxapampa, 10°42.2'S, 75°28.1'W, 10.02.2011, H = 1977 m, leg. Viktor Sinyaev & Alexander Poleschuk / UF, FLMNH, MGCL 1049179. [DNA voucher MGCL-NOC- 65328] deposited in MGCL. ***Paratypes*** (6 ♂, MGCL): **Peru**: Peru, Dept. Junin, Cerro Pichita, Res. Sta. nr. San Ramon, 2165 m, 7–9 Apr. 2011, coll. J. B. Heppner & C. Carrera (2 ♂); Peru, Department Cuzco, Manu Park, San Pedro, 1800 m, Mar. 1997, coll. local people (3 ♂); **Bolivia**: Bolivia, La Paz, Cotapata, 16°16.5'S, 67°51.6'W, 24.10.2010, H = 3200 m, leg. Viktor Sinyaev & Oleg Romanov (1 ♂).

##### Etymology.

The name of this species is in honor of my colleague and friend Dr. B. Christian Schmidt, who shared his knowledge with me about Noctuoidea without hesitation.

##### Diagnosis.

*Oculicattusschmidti* has a set of remarkable characters that separate it from other species, such as the orbicular spot is rounded; the lunate marking of the reniform spot is incomplete, being similar to those of *Gaujonia* species; the forewing is more stylized with straighter margins. The male genitalia are similar to those of *O.boliviana*, but can be distinguished from them by the cucullar region, which is narrower than those of other species; the saccular is large and densely covered with setae; also the uncus is wider and the saccus longer than the other species of *Oculicattus*. The DNA barcode is similar to that of *O.renifera*; however, external and internal morphology reveal enough differences to identify the two species.

##### Description.

***Head*.** Palp marbled in black and white with frons yellowish gray; black patch between antennae small. ***Thorax*.** Covered in yellow with some black patches on dorsum. ***Wing*.** Forewing length: male 17–19 mm; forewing, dark yellow with well-developed lines from posterior margin to fold that look similar to those of other *Oculicattus* species; reniform spot with lunate marking narrowed in middle of base of cell M1; orbicular spot small, outlined in black, rounded; V-shaped mark on CuA2 base small, with upper line longer, extending to lower side of reniform spot; hindwing with fringe marbled in yellow and black; posterior margin with a combination of yellow and brown hair-like scales; veins yellow with two black lines in middle of veins from M2 to CuA2; black line posterior to base of CuA2. ***Leg.*** Prothoracic and mesothoracic legs marbled in yellow and black; metathoracic legs yellow. ***Abdomen*.** Dark yellow with dorsum clothed with dark brown and black scales; yellow tufts in middle of A1–A4 with a small vertical line on each tuft. ***Male genitalia*.** Cucullus bullhorn-like, with pointed apex heavily coated with setae; sacculus narrow with its process long and wide, densely covered with setae; terminus of saccular process tapered to apex; saccus long rhomboid-shaped, tip barely rounded; juxta shield-shaped and upper side concave; juxta with an expanded upper side; wide tegumen; aedeagus ¼ × longer than basal area of vesica, remarkable curved inwards; vesica ⅔ × longer than wide; one of patches of spines on tip small slightly squared and other covering ⅓ of whole vesica.

##### Immature stages.

Unknown.

##### Distribution.

This species is distributed from Central region of Peru and to the east region of Bolivia (Fig. 95).

##### Biology.

Unknown.

##### Remarks.

Holotype in perfect condition (Fig. 45), paratypes in good condition even though the forewings are slightly damaged, but they are still well preserved.

#### Oculicattus uturunku sp. nov.

Taxon classificationAnimaliaLepidopteraNoctuidae

F3C8831A-B254-5931-9593-32D2CB89819C

http://zoobank.org/6C34E855-780E-4A8F-9BF5-C1E39FE26236

Figs 49 , 76 , 95

##### Type material.

***Holotype*** ♂, **Ecuador**: ECUADOR, Morona, Santiago 9km. Road Plan de Milagro – Gualaceo, 3°00'04"S, 78°30'49"W, 15.02.2012, H = 2375m, Exped. Ron Brechlin & Victor Sinyaev / FLMNH, MGCL 1049085. [DNA voucher MGCL-NOC- 65266] deposited in MGCL. ***Paratypes*** (3 ♂, MGCL): **Ecuador**: ECUADOR, CARCHI Prov., El Angel Ecological Reserve, 0°45'31"N, 78°01'40"W, 7–8.11.2012, H = 3320 m, Exped. Ron Brechlin & Victor Sinyaev (1 ♂); ECUADOR, Carchi prov., El Moran, 0°45'50"N, 78°02'38"W, 1–3.05.2012, H = 2940 m, Exped. Ron Brechlin & Victor Sinyaev (1 ♂); ECUADOR, CARCHI Prov., El Chical – Carolinae0°50'20"N, 78°13'39"W, 20.11.2012, H = 2360 m, Exped. Ron Brechlin & Victor Sinyaev (1 ♂).

##### Etymology.

The name *uturunku* makes reference to the jaguar *Pantheraonca* (Linnaeus) in the Quechua language.

##### Diagnosis.

There is only one species that has similar characters to *O.uturunku*, which is *O.raizae*. Nevertheless, they are easy to separate because the color black is predominant in *O.uturunku* and the yellow is secondary. The reniform spot is black and quite smaller, converging with long black lines making it look like an eyelash. Genitalia of the male are remarkably smaller than *O.raizae*, mainly the cucullus, which is also remarkably narrower.

##### Description.

***Head*.** Palp with last segment divided into three parts, base and tip yellow and middle area black; frons dark yellow, with a large black band between antennae. ***Thorax*.** Marbled in black and sulfur-yellow dorsally, and sulfur-yellow ventrally. ***Wing*.** Forewing length, male 20–22 mm; forewing black with some regions of sulfur-yellow; enormous black lines on veins define forewing pattern; black orbicular spot small and elongated; unusual reniform spot eyelash-like, black; black line through inferior region of discal cell barely touching base of CuA2; hindwing with black fringe and some sulfur-yellow scales at end of each vein paler than forewing, whereas that from posterior margin completely gray; veins black with some spots of sulfur-yellow. ***Leg.*** Prothoracic and mesothoracic legs black with some sulfur-yellow on joints, and metathoracic legs in yellow. ***Abdomen*.** Black with segments sulfur-yellow ventrally, whereas dorsally dark gray, paler on first three segments; A1–A3 with tufts in yellow and with some black scales. ***Male genitalia*.** Cucullus wider on base and apex small; costal margin curved; sacculus and process wide; saccus narrow and rhomboid-shaped; juxta square-shaped with base narrower; tegumen narrow; aedeagus 3 × longer than wide; basal area of vesica 1 ½ × longer than vesica itself; large slightly curved patch of spines close to basal area; one of patches of spines on tip small with triangular terminus and another larger covering almost ½ of vesica.

##### Immature stages.

Unknown.

##### Distribution.

*Oculicattusuturunku* occurs mainly in the Western Cordillera of the Andes in Ecuador. It is found at high to very high elevations (Fig. 95).

##### Biology.

Unknown.

##### Remarks.

Holotype (Fig. 49) and paratypes in good condition. The species *Oculicattusuturunku* has been confused with Gaujonianr.renifera by Piñas et al. (2002). The DNA barcode is very similar to *O.schmidti* (see *O.schmidti* diagnosis)

**Figure 91.**
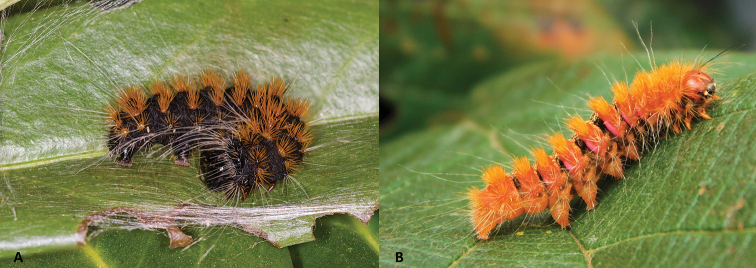
**A, B** seventh instar larva **A***Cicadomorphusfalkasiska*, Oxapampa, Peru **B***Gaujoniakanakusika* Cundinamarca, Colombia.

**Figure 92.**
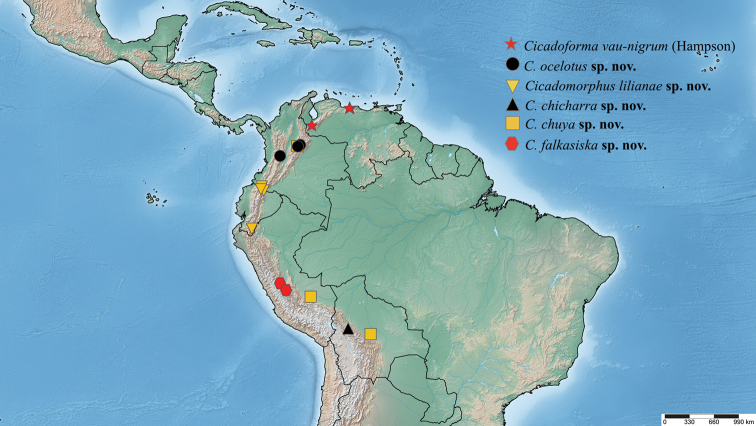
Distribution of examined specimens of the genera *Cicadoforma* and *Cicadomorphus*.

**Figure 93.**
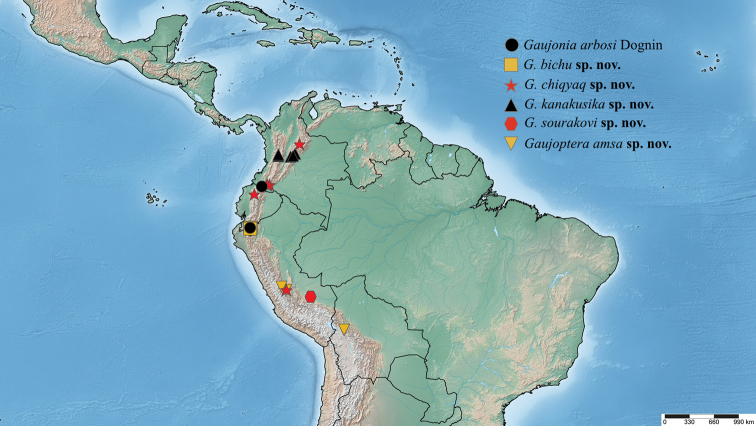
Distribution of examined specimens of the genera *Gaujonia* and *Gaujoptera*.

**Figure 94.**
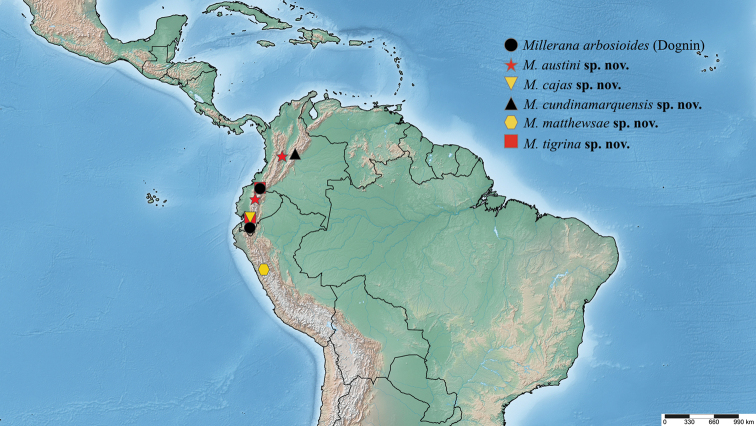
Distribution of examined specimens of the genus *Millerana*.

**Figure 95.**
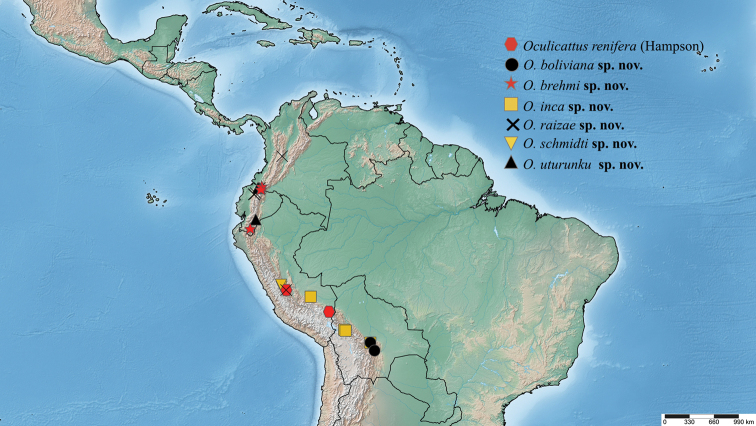
Distribution of examined specimens of the genus *Oculicattus*.

## Discussion

The Andean genus *Gaujonia* had not been properly studied since its discovery more than a century ago (Dognin 1891), and thus its life cycle and taxonomic status, were essentially unknown. Nevertheless, during this revision, I found that *Gaujonia* was not a monophyletic group, but included species representing five genera. It is worth mentioning that the whole generic complex showed signs of mtDNA introgression or introgressive hybridization, found by studying Cytochrome c oxidase subunit I (COI), which has been observed in other groups of noctuoids even in other member of the subfamily Pantheinae (Schmidt and Sperling 2008; Anweiler 2009; Otim et al. 2018, Schmidt and Anweiler 2020).

The above morphological characters and the DNA barcode data demonstrated that the members of the *Gaujonia* genus group are sister to each other, but surprisingly despite similarities between the *renifera* and *arbosi* groups, the *arbosi* and *vau-nigrum* groups were more closely related; thus, all four groups were separated into different genera. The *arbosi* group is established now as the true *Gaujonia* due to significant differences with the other groups. Three new genera, *Millerana* gen. nov., *Oculicattus* gen. nov., and *Cicadoforma* gen. nov., are proposed for *arbosioides*, *renifera*, and *vau-nigrum* groups, respectively. In addition, there were other specimens that were misidentified as *Gaujonia* sp., but molecular and morphological analyses demonstrated that they belong to different genera (*Gaujoptera* gen. nov. and *Cicadoforma* gen. nov.). *Gaujoptera* gen. nov. was found to be closely related to the genus *Millerana* and the *Gaujonia* generic group, whereas *Cicadomorphus* gen. nov. is close to *Cicadoforma* gen. nov. In addition, the species *Gaujoniakanakusika* proved to be genetically different from the other species in the genus *Gaujonia*, however, the apomorphic characters observed in this species were not sufficient to separate this species into a different genus.

On the other hand, it was found that the *Gaujonia* genus group is represented by cryptic genera, which have similar morphology but are genetically distant, such as *Gaujonia* and *Oculicattus*, as well as *Cicadoforma* and *Cicadomorphus*. However, the same problem was observed with the molecular characters in some species groups: *Gaujoniaarbosi*–*G.chiqyaq*, *Oculicattusboliviana*–*O.uturunku*, and *O.schmidti*–*O.renifera*, which showed slight differences in the COI sequences. Therefore, both morphological and molecular characters played a critical role to make an appropriate identification, not only in this genus group, but in general since there are many cryptic genera and species that are still misidentified or even undiscovered.

In addition, despite what was proposed by Schmidt and Anweiler (2020) about the closer relationship between *Arctioptera* Schmidt & Anweiler and the rest of the jaguar moths based on similar external morphology, the phylogenetic analysis placed *Arctioptera* closer to the genera *Meleneta* Smith and *Charadra* Walker. The internal morphology also shows more similar characters with the genus *Colocasia* than with the jaguar moths.

Another important discovery was that some endemic species (*Cicadomorphusfalkasiska* and *Gaujoniakanakusika*) are specialists on at-risk plants such as *Alnusacuminata* Kunth and *Prunussubcorymbosa* Ruiz ex Koehne and thus are themselves vulnerable to extinction (IUCN 2019). Additionally, these species along with others, are restricted to small areas. Therefore, the group is an ideal target for conservation efforts.

Finally, it was found that larvae of *Cicadomorphusfalkasiska* regurgitate a transparent highly alkaline chemical compound, which was observed to deter ants (JIM pers. obs.).

## Supplementary Material

XML Treatment for Cicadoforma

XML Treatment for Cicadoforma ocelotus

XML Treatment for Cicadoforma vau-nigrum

XML Treatment for Cicadomorphus

XML Treatment for Cicadomorphus chicharra

XML Treatment for Cicadomorphus chuya

XML Treatment for Cicadomorphus falkasiska

XML Treatment for Cicadomorphus lilianae

XML Treatment for Gaujonia

XML Treatment for Gaujonia arbosi

XML Treatment for Gaujonia bichu

XML Treatment for Gaujonia chiqyaq

XML Treatment for Gaujonia kanakusika

XML Treatment for Gaujonia sourakovi

XML Treatment for Gaujoptera

XML Treatment for Gaujoptera amsa

XML Treatment for Millerana

XML Treatment for Millerana arbosioides

XML Treatment for Millerana austini

XML Treatment for Millerana cajas

XML Treatment for Millerana cundinamarquensis

XML Treatment for Millerana matthewsae

XML Treatment for Millerana tigrina

XML Treatment for Oculicattus

XML Treatment for Oculicattus boliviana

XML Treatment for Oculicattus brehmi

XML Treatment for Oculicattus inca

XML Treatment for Oculicattus raizae

XML Treatment for Oculicattus renifera

XML Treatment for Oculicattus schmidti

XML Treatment for Oculicattus uturunku

## Supplementary materials

Supplementary material 1 Table S1 Data type genetic data Explanation note Sequences information employed in this study available at GenBank (http://www.ncbi.nlm.nih.gov/Genbank) and BARCODE OF LIFE DATA SYSTEM v4 (http://barcodinglife.com). https://binary.pensoft.net/file/471618This dataset is made available under the Open Database License (http://opendatacommons.org/licenses/odbl/1.0/). The Open Database License (ODbL) is a license agreement intended to allow users to freely share, modify, and use this Dataset while maintaining this same freedom for others, provided that the original source and author(s) are credited.Jose I. Martinez

Supplementary material 2 Figure S1 Data type image Explanation note Phylogeny of *Gaujonia* and related genera based on a maximum likelihood analysis of a mitochondrial cytochrome oxidase I (COI) dataset. https://binary.pensoft.net/file/471619This dataset is made available under the Open Database License (http://opendatacommons.org/licenses/odbl/1.0/). The Open Database License (ODbL) is a license agreement intended to allow users to freely share, modify, and use this Dataset while maintaining this same freedom for others, provided that the original source and author(s) are credited.Jose I. Martinez

Supplementary material 3 File S1 Data type sequencing data Explanation note FASTA file containing the alignment of all COI sequences of the taxa used in the Figure 1 and Figure S1. https://binary.pensoft.net/file/471620This dataset is made available under the Open Database License (http://opendatacommons.org/licenses/odbl/1.0/). The Open Database License (ODbL) is a license agreement intended to allow users to freely share, modify, and use this Dataset while maintaining this same freedom for others, provided that the original source and author(s) are credited.Jose I. Martinez

Supplementary material 4 File S2 Data type phylogenetic data Explanation note Tree file in NEWICK format correlated with the gene tree in the Figure 1 and Figure S1. https://binary.pensoft.net/file/471621This dataset is made available under the Open Database License (http://opendatacommons.org/licenses/odbl/1.0/). The Open Database License (ODbL) is a license agreement intended to allow users to freely share, modify, and use this Dataset while maintaining this same freedom for others, provided that the original source and author(s) are credited.Jose I. Martinez
