# Supplementary figures and images for: Revision of the South American genus Gaujonia Dognin (Noctuidae, Pantheinae) with descriptions of five new genera and twenty-one new species
Source: Zookeys. 2020 Nov 5;985:71–126. doi: 10.3897/zookeys.985.51622 (PMC7661952; doi:10.3897/zookeys.985.51622)

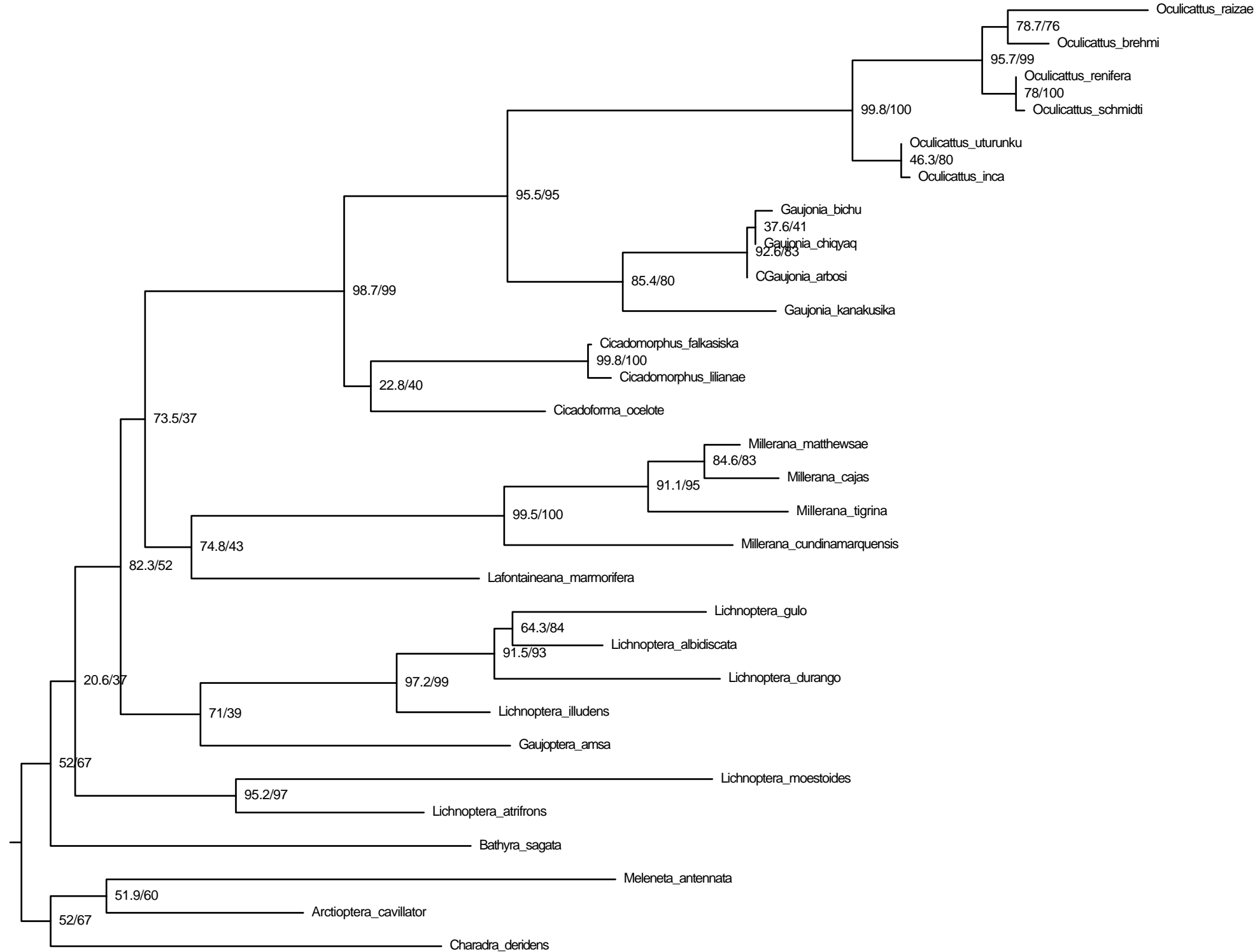

0.03

Supplement: Supplementary material 2 — Figure S1 [file zookeys-985-071-s002.pdf]
